# Measurement of the centrality and pseudorapidity dependence of the integrated elliptic flow in lead–lead collisions at $\sqrt{s_{\mathrm {NN}}}=2.76$ TeV with the ATLAS detector

**DOI:** 10.1140/epjc/s10052-014-2982-4

**Published:** 2014-08-13

**Authors:** The ATLAS Collaboration, G. Aad, B. Abbott, J. Abdallah, S. Abdel Khalek, O. Abdinov, R. Aben, B. Abi, M. Abolins, O. S. AbouZeid, H. Abramowicz, H. Abreu, R. Abreu, Y. Abulaiti, B. S. Acharya, L. Adamczyk, D. L. Adams, J. Adelman, S. Adomeit, T. Adye, T. Agatonovic-Jovin, J. A. Aguilar-Saavedra, M. Agustoni, S. P. Ahlen, F. Ahmadov, G. Aielli, T. P. A. Åkesson, G. Akimoto, A. V. Akimov, G. L. Alberghi, J. Albert, S. Albrand, M. J. Alconada Verzini, M. Aleksa, I. N. Aleksandrov, C. Alexa, G. Alexander, G. Alexandre, T. Alexopoulos, M. Alhroob, G. Alimonti, L. Alio, J. Alison, B. M. M. Allbrooke, L. J. Allison, P. P. Allport, S. E. Allwood-Spiers, J. Almond, A. Aloisio, A. Alonso, F. Alonso, C. Alpigiani, A. Altheimer, B. Alvarez Gonzalez, M. G. Alviggi, K. Amako, Y. Amaral Coutinho, C. Amelung, D. Amidei, S. P. Amor Dos Santos, A. Amorim, S. Amoroso, N. Amram, G. Amundsen, C. Anastopoulos, L. S. Ancu, N. Andari, T. Andeen, C. F. Anders, G. Anders, K. J. Anderson, A. Andreazza, V. Andrei, X. S. Anduaga, S. Angelidakis, I. Angelozzi, P. Anger, A. Angerami, F. Anghinolfi, A. V. Anisenkov, N. Anjos, A. Annovi, A. Antonaki, M. Antonelli, A. Antonov, J. Antos, F. Anulli, M. Aoki, L. Aperio Bella, R. Apolle, G. Arabidze, I. Aracena, Y. Arai, J. P. Araque, A. T. H. Arce, J-F. Arguin, S. Argyropoulos, M. Arik, A. J. Armbruster, O. Arnaez, V. Arnal, H. Arnold, O. Arslan, A. Artamonov, G. Artoni, S. Asai, N. Asbah, A. Ashkenazi, S. Ask, B. Åsman, L. Asquith, K. Assamagan, R. Astalos, M. Atkinson, N. B. Atlay, B. Auerbach, K. Augsten, M. Aurousseau, G. Avolio, G. Azuelos, Y. Azuma, M. A. Baak, C. Bacci, H. Bachacou, K. Bachas, M. Backes, M. Backhaus, J. Backus Mayes, E. Badescu, P. Bagiacchi, P. Bagnaia, Y. Bai, T. Bain, J. T. Baines, O. K. Baker, S. Baker, P. Balek, F. Balli, E. Banas, Sw. Banerjee, A. Bangert, A. A. E. Bannoura, V. Bansal, H. S. Bansil, L. Barak, S. P. Baranov, E. L. Barberio, D. Barberis, M. Barbero, T. Barillari, M. Barisonzi, T. Barklow, N. Barlow, B. M. Barnett, R. M. Barnett, Z. Barnovska, A. Baroncelli, G. Barone, A. J. Barr, F. Barreiro, J. Barreiro Guimarães da Costa, R. Bartoldus, A. E. Barton, P. Bartos, V. Bartsch, A. Bassalat, A. Basye, R. L. Bates, L. Batkova, J. R. Batley, M. Battistin, F. Bauer, H. S. Bawa, T. Beau, P. H. Beauchemin, R. Beccherle, P. Bechtle, H. P. Beck, K. Becker, S. Becker, M. Beckingham, C. Becot, A. J. Beddall, A. Beddall, S. Bedikian, V. A. Bednyakov, C. P. Bee, L. J. Beemster, T. A. Beermann, M. Begel, K. Behr, C. Belanger-Champagne, P. J. Bell, W. H. Bell, G. Bella, L. Bellagamba, A. Bellerive, M. Bellomo, A. Belloni, K. Belotskiy, O. Beltramello, O. Benary, D. Benchekroun, K. Bendtz, N. Benekos, Y. Benhammou, E. Benhar Noccioli, J. A. Benitez Garcia, D. P. Benjamin, J. R. Bensinger, K. Benslama, S. Bentvelsen, D. Berge, E. Bergeaas Kuutmann, N. Berger, F. Berghaus, E. Berglund, J. Beringer, C. Bernard, P. Bernat, C. Bernius, F. U. Bernlochner, T. Berry, P. Berta, C. Bertella, F. Bertolucci, M. I. Besana, G. J. Besjes, O. Bessidskaia, N. Besson, C. Betancourt, S. Bethke, W. Bhimji, R. M. Bianchi, L. Bianchini, M. Bianco, O. Biebel, S. P. Bieniek, K. Bierwagen, J. Biesiada, M. Biglietti, J. Bilbao De Mendizabal, H. Bilokon, M. Bindi, S. Binet, A. Bingul, C. Bini, C. W. Black, J. E. Black, K. M. Black, D. Blackburn, R. E. Blair, J.-B. Blanchard, T. Blazek, I. Bloch, C. Blocker, W. Blum, U. Blumenschein, G. J. Bobbink, V. S. Bobrovnikov, S. S. Bocchetta, A. Bocci, C. R. Boddy, M. Boehler, J. Boek, T. T. Boek, J. A. Bogaerts, A. G. Bogdanchikov, A. Bogouch, C. Bohm, J. Bohm, V. Boisvert, T. Bold, V. Boldea, A. S. Boldyrev, M. Bomben, M. Bona, M. Boonekamp, A. Borisov, G. Borissov, M. Borri, S. Borroni, J. Bortfeldt, V. Bortolotto, K. Bos, D. Boscherini, M. Bosman, H. Boterenbrood, J. Boudreau, J. Bouffard, E. V. Bouhova-Thacker, D. Boumediene, C. Bourdarios, N. Bousson, S. Boutouil, A. Boveia, J. Boyd, I. R. Boyko, I. Bozovic-Jelisavcic, J. Bracinik, P. Branchini, A. Brandt, G. Brandt, O. Brandt, U. Bratzler, B. Brau, J. E. Brau, H. M. Braun, S. F. Brazzale, B. Brelier, K. Brendlinger, A. J. Brennan, R. Brenner, S. Bressler, K. Bristow, T. M. Bristow, D. Britton, F. M. Brochu, I. Brock, R. Brock, C. Bromberg, J. Bronner, G. Brooijmans, T. Brooks, W. K. Brooks, J. Brosamer, E. Brost, G. Brown, J. Brown, P. A. Bruckman de Renstrom, D. Bruncko, R. Bruneliere, S. Brunet, A. Bruni, G. Bruni, M. Bruschi, L. Bryngemark, T. Buanes, Q. Buat, F. Bucci, P. Buchholz, R. M. Buckingham, A. G. Buckley, S. I. Buda, I. A. Budagov, F. Buehrer, L. Bugge, M. K. Bugge, O. Bulekov, A. C. Bundock, H. Burckhart, S. Burdin, B. Burghgrave, S. Burke, I. Burmeister, E. Busato, D. Büscher, V. Büscher, P. Bussey, C. P. Buszello, B. Butler, J. M. Butler, A. I. Butt, C. M. Buttar, J. M. Butterworth, P. Butti, W. Buttinger, A. Buzatu, M. Byszewski, S. Cabrera Urbán, D. Caforio, O. Cakir, P. Calafiura, A. Calandri, G. Calderini, P. Calfayan, R. Calkins, L. P. Caloba, D. Calvet, S. Calvet, R. Camacho Toro, S. Camarda, D. Cameron, L. M. Caminada, R. Caminal Armadans, S. Campana, M. Campanelli, A. Campoverde, V. Canale, A. Canepa, J. Cantero, R. Cantrill, T. Cao, M. D. M. Capeans Garrido, I. Caprini, M. Caprini, M. Capua, R. Caputo, R. Cardarelli, T. Carli, G. Carlino, L. Carminati, S. Caron, E. Carquin, G. D. Carrillo-Montoya, J. R. Carter, J. Carvalho, D. Casadei, M. P. Casado, M. Casolino, E. Castaneda-Miranda, A. Castelli, V. Castillo Gimenez, N. F. Castro, P. Catastini, A. Catinaccio, J. R. Catmore, A. Cattai, G. Cattani, S. Caughron, V. Cavaliere, D. Cavalli, M. Cavalli-Sforza, V. Cavasinni, F. Ceradini, B. Cerio, K. Cerny, A. S. Cerqueira, A. Cerri, L. Cerrito, F. Cerutti, M. Cerv, A. Cervelli, S. A. Cetin, A. Chafaq, D. Chakraborty, I. Chalupkova, K. Chan, P. Chang, B. Chapleau, J. D. Chapman, D. Charfeddine, D. G. Charlton, C. C. Chau, C. A. Chavez Barajas, S. Cheatham, A. Chegwidden, S. Chekanov, S. V. Chekulaev, G. A. Chelkov, M. A. Chelstowska, C. Chen, H. Chen, K. Chen, L. Chen, S. Chen, X. Chen, Y. Chen, H. C. Cheng, Y. Cheng, A. Cheplakov, R. Cherkaoui El Moursli, V. Chernyatin, E. Cheu, L. Chevalier, V. Chiarella, G. Chiefari, J. T. Childers, A. Chilingarov, G. Chiodini, A. S. Chisholm, R. T. Chislett, A. Chitan, M. V. Chizhov, S. Chouridou, B. K. B. Chow, I. A. Christidi, D. Chromek-Burckhart, M. L. Chu, J. Chudoba, J. J. Chwastowski, L. Chytka, G. Ciapetti, A. K. Ciftci, R. Ciftci, D. Cinca, V. Cindro, A. Ciocio, P. Cirkovic, Z. H. Citron, M. Citterio, M. Ciubancan, A. Clark, P. J. Clark, R. N. Clarke, W. Cleland, J. C. Clemens, C. Clement, Y. Coadou, M. Cobal, A. Coccaro, J. Cochran, L. Coffey, J. G. Cogan, J. Coggeshall, B. Cole, S. Cole, A. P. Colijn, C. Collins-Tooth, J. Collot, T. Colombo, G. Colon, G. Compostella, P. Conde Muiño, E. Coniavitis, M. C. Conidi, S. H. Connell, I. A. Connelly, S. M. Consonni, V. Consorti, S. Constantinescu, C. Conta, G. Conti, F. Conventi, M. Cooke, B. D. Cooper, A. M. Cooper-Sarkar, N. J. Cooper-Smith, K. Copic, T. Cornelissen, M. Corradi, F. Corriveau, A. Corso-Radu, A. Cortes-Gonzalez, G. Cortiana, G. Costa, M. J. Costa, D. Costanzo, D. Côté, G. Cottin, G. Cowan, B. E. Cox, K. Cranmer, G. Cree, S. Crépé-Renaudin, F. Crescioli, M. Crispin Ortuzar, M. Cristinziani, V. Croft, G. Crosetti, C.-M. Cuciuc, T. Cuhadar Donszelmann, J. Cummings, M. Curatolo, C. Cuthbert, H. Czirr, P. Czodrowski, Z. Czyczula, S. D’Auria, M. D’Onofrio, M. J. Da Cunha Sargedas De Sousa, C. Da Via, W. Dabrowski, A. Dafinca, T. Dai, O. Dale, F. Dallaire, C. Dallapiccola, M. Dam, A. C. Daniells, M. Dano Hoffmann, V. Dao, G. Darbo, G. L. Darlea, S. Darmora, J. A. Dassoulas, A. Dattagupta, W. Davey, C. David, T. Davidek, E. Davies, M. Davies, O. Davignon, A. R. Davison, P. Davison, Y. Davygora, E. Dawe, I. Dawson, R. K. Daya-Ishmukhametova, K. De, R. de Asmundis, S. De Castro, S. De Cecco, J. de Graat, N. De Groot, P. de Jong, H. De la Torre, F. De Lorenzi, L. De Nooij, D. De Pedis, A. De Salvo, U. De Sanctis, A. De Santo, J. B. De Vivie De Regie, G. De Zorzi, W. J. Dearnaley, R. Debbe, C. Debenedetti, B. Dechenaux, D. V. Dedovich, J. Degenhardt, I. Deigaard, J. Del Peso, T. Del Prete, F. Deliot, C. M. Delitzsch, M. Deliyergiyev, A. Dell’Acqua, L. Dell’Asta, M. Dell’Orso, M. Della Pietra, D. della Volpe, M. Delmastro, P. A. Delsart, C. Deluca, S. Demers, M. Demichev, A. Demilly, S. P. Denisov, D. Derendarz, J. E. Derkaoui, F. Derue, P. Dervan, K. Desch, C. Deterre, P. O. Deviveiros, A. Dewhurst, S. Dhaliwal, A. Di Ciaccio, L. Di Ciaccio, A. Di Domenico, C. Di Donato, A. Di Girolamo, B. Di Girolamo, A. Di Mattia, B. Di Micco, R. Di Nardo, A. Di Simone, R. Di Sipio, D. Di Valentino, M. A. Diaz, E. B. Diehl, J. Dietrich, T. A. Dietzsch, S. Diglio, A. Dimitrievska, J. Dingfelder, C. Dionisi, P. Dita, S. Dita, F. Dittus, F. Djama, T. Djobava, M. A. B. do Vale, A. Do Valle Wemans, T. K. O. Doan, D. Dobos, E. Dobson, C. Doglioni, T. Doherty, T. Dohmae, J. Dolejsi, Z. Dolezal, B. A. Dolgoshein, M. Donadelli, S. Donati, P. Dondero, J. Donini, J. Dopke, A. Doria, M. T. Dova, A. T. Doyle, M. Dris, J. Dubbert, S. Dube, E. Dubreuil, E. Duchovni, G. Duckeck, O. A. Ducu, D. Duda, A. Dudarev, F. Dudziak, L. Duflot, L. Duguid, M. Dührssen, M. Dunford, H. Duran Yildiz, M. Düren, A. Durglishvili, M. Dwuznik, M. Dyndal, J. Ebke, W. Edson, N. C. Edwards, W. Ehrenfeld, T. Eifert, G. Eigen, K. Einsweiler, T. Ekelof, M. El Kacimi, M. Ellert, S. Elles, F. Ellinghaus, N. Ellis, J. Elmsheuser, M. Elsing, D. Emeliyanov, Y. Enari, O. C. Endner, M. Endo, R. Engelmann, J. Erdmann, A. Ereditato, D. Eriksson, G. Ernis, J. Ernst, M. Ernst, J. Ernwein, D. Errede, S. Errede, E. Ertel, M. Escalier, H. Esch, C. Escobar, B. Esposito, A. I. Etienvre, E. Etzion, H. Evans, L. Fabbri, G. Facini, R. M. Fakhrutdinov, S. Falciano, J. Faltova, Y. Fang, M. Fanti, A. Farbin, A. Farilla, T. Farooque, S. Farrell, S. M. Farrington, P. Farthouat, F. Fassi, P. Fassnacht, D. Fassouliotis, A. Favareto, L. Fayard, P. Federic, O. L. Fedin, W. Fedorko, M. Fehling-Kaschek, S. Feigl, L. Feligioni, C. Feng, E. J. Feng, H. Feng, A. B. Fenyuk, S. Fernandez Perez, S. Ferrag, J. Ferrando, A. Ferrari, P. Ferrari, R. Ferrari, D. E. Ferreira de Lima, A. Ferrer, D. Ferrere, C. Ferretti, A. Ferretto Parodi, M. Fiascaris, F. Fiedler, A. Filipčič, M. Filipuzzi, F. Filthaut, M. Fincke-Keeler, K. D. Finelli, M. C. N. Fiolhais, L. Fiorini, A. Firan, J. Fischer, W. C. Fisher, E. A. Fitzgerald, M. Flechl, I. Fleck, P. Fleischmann, S. Fleischmann, G. T. Fletcher, G. Fletcher, T. Flick, A. Floderus, L. R. Flores Castillo, A. C. Florez Bustos, M. J. Flowerdew, A. Formica, A. Forti, D. Fortin, D. Fournier, H. Fox, S. Fracchia, P. Francavilla, M. Franchini, S. Franchino, D. Francis, M. Franklin, S. Franz, M. Fraternali, S. T. French, C. Friedrich, F. Friedrich, D. Froidevaux, J. A. Frost, C. Fukunaga, E. Fullana Torregrosa, B. G. Fulsom, J. Fuster, C. Gabaldon, O. Gabizon, A. Gabrielli, A. Gabrielli, S. Gadatsch, S. Gadomski, G. Gagliardi, P. Gagnon, C. Galea, B. Galhardo, E. J. Gallas, V. Gallo, B. J. Gallop, P. Gallus, G. Galster, K. K. Gan, R. P. Gandrajula, J. Gao, Y. S. Gao, F. M. Garay Walls, F. Garberson, C. García, J. E. García Navarro, M. Garcia-Sciveres, R. W. Gardner, N. Garelli, V. Garonne, C. Gatti, G. Gaudio, B. Gaur, L. Gauthier, P. Gauzzi, I. L. Gavrilenko, C. Gay, G. Gaycken, E. N. Gazis, P. Ge, Z. Gecse, C. N. P. Gee, D. A. A. Geerts, Ch. Geich-Gimbel, K. Gellerstedt, C. Gemme, A. Gemmell, M. H. Genest, S. Gentile, M. George, S. George, D. Gerbaudo, A. Gershon, H. Ghazlane, N. Ghodbane, B. Giacobbe, S. Giagu, V. Giangiobbe, P. Giannetti, F. Gianotti, B. Gibbard, S. M. Gibson, M. Gilchriese, T. P. S. Gillam, D. Gillberg, G. Gilles, D. M. Gingrich, N. Giokaris, M. P. Giordani, R. Giordano, F. M. Giorgi, P. F. Giraud, D. Giugni, C. Giuliani, M. Giulini, B. K. Gjelsten, I. Gkialas, L. K. Gladilin, C. Glasman, J. Glatzer, P. C. F. Glaysher, A. Glazov, G. L. Glonti, M. Goblirsch-Kolb, J. R. Goddard, J. Godfrey, J. Godlewski, C. Goeringer, S. Goldfarb, T. Golling, D. Golubkov, A. Gomes, L. S. Gomez Fajardo, R. Gonçalo, J. Goncalves Pinto Firmino Da Costa, L. Gonella, S. González de la Hoz, G. Gonzalez Parra, M. L. Gonzalez Silva, S. Gonzalez-Sevilla, L. Goossens, P. A. Gorbounov, H. A. Gordon, I. Gorelov, B. Gorini, E. Gorini, A. Gorišek, E. Gornicki, A. T. Goshaw, C. Gössling, M. I. Gostkin, M. Gouighri, D. Goujdami, M. P. Goulette, A. G. Goussiou, C. Goy, S. Gozpinar, H. M. X. Grabas, L. Graber, I. Grabowska-Bold, P. Grafström, K-J. Grahn, J. Gramling, E. Gramstad, S. Grancagnolo, V. Grassi, V. Gratchev, H. M. Gray, E. Graziani, O. G. Grebenyuk, Z. D. Greenwood, K. Gregersen, I. M. Gregor, P. Grenier, J. Griffiths, A. A. Grillo, K. Grimm, S. Grinstein, Ph. Gris, Y. V. Grishkevich, J-F. Grivaz, J. P. Grohs, A. Grohsjean, E. Gross, J. Grosse-Knetter, G. C. Grossi, J. Groth-Jensen, Z. J. Grout, K. Grybel, L. Guan, F. Guescini, D. Guest, O. Gueta, C. Guicheney, E. Guido, T. Guillemin, S. Guindon, U. Gul, C. Gumpert, J. Gunther, J. Guo, S. Gupta, P. Gutierrez, N. G. Gutierrez Ortiz, C. Gutschow, N. Guttman, C. Guyot, C. Gwenlan, C. B. Gwilliam, A. Haas, C. Haber, H. K. Hadavand, N. Haddad, P. Haefner, S. Hageboeck, Z. Hajduk, H. Hakobyan, M. Haleem, D. Hall, G. Halladjian, K. Hamacher, P. Hamal, K. Hamano, M. Hamer, A. Hamilton, S. Hamilton, P. G. Hamnett, L. Han, K. Hanagaki, K. Hanawa, M. Hance, P. Hanke, J. B. Hansen, J. D. Hansen, P. H. Hansen, K. Hara, A. S. Hard, T. Harenberg, S. Harkusha, D. Harper, R. D. Harrington, O. M. Harris, P. F. Harrison, F. Hartjes, S. Hasegawa, Y. Hasegawa, A. Hasib, S. Hassani, S. Haug, M. Hauschild, R. Hauser, M. Havranek, C. M. Hawkes, R. J. Hawkings, A. D. Hawkins, T. Hayashi, D. Hayden, C. P. Hays, H. S. Hayward, S. J. Haywood, S. J. Head, T. Heck, V. Hedberg, L. Heelan, S. Heim, T. Heim, B. Heinemann, L. Heinrich, S. Heisterkamp, J. Hejbal, L. Helary, C. Heller, M. Heller, S. Hellman, D. Hellmich, C. Helsens, J. Henderson, R. C. W. Henderson, C. Hengler, A. Henrichs, A. M. Henriques Correia, S. Henrot-Versille, C. Hensel, G. H. Herbert, Y. Hernández Jiménez, R. Herrberg-Schubert, G. Herten, R. Hertenberger, L. Hervas, G. G. Hesketh, N. P. Hessey, R. Hickling, E. Higón-Rodriguez, E. Hill, J. C. Hill, K. H. Hiller, S. Hillert, S. J. Hillier, I. Hinchliffe, E. Hines, M. Hirose, D. Hirschbuehl, J. Hobbs, N. Hod, M. C. Hodgkinson, P. Hodgson, A. Hoecker, M. R. Hoeferkamp, J. Hoffman, D. Hoffmann, J. I. Hofmann, M. Hohlfeld, T. R. Holmes, T. M. Hong, L. Hooft van Huysduynen, J-Y. Hostachy, S. Hou, A. Hoummada, J. Howard, J. Howarth, M. Hrabovsky, I. Hristova, J. Hrivnac, T. Hryn’ova, P. J. Hsu, S.-C. Hsu, D. Hu, X. Hu, Y. Huang, Z. Hubacek, F. Hubaut, F. Huegging, T. B. Huffman, E. W. Hughes, G. Hughes, M. Huhtinen, T. A. Hülsing, M. Hurwitz, N. Huseynov, J. Huston, J. Huth, G. Iacobucci, G. Iakovidis, I. Ibragimov, L. Iconomidou-Fayard, E. Ideal, P. Iengo, O. Igonkina, T. Iizawa, Y. Ikegami, K. Ikematsu, M. Ikeno, D. Iliadis, N. Ilic, Y. Inamaru, T. Ince, P. Ioannou, M. Iodice, K. Iordanidou, V. Ippolito, A. Irles Quiles, C. Isaksson, M. Ishino, M. Ishitsuka, R. Ishmukhametov, C. Issever, S. Istin, J. M. Iturbe Ponce, J. Ivarsson, A. V. Ivashin, W. Iwanski, H. Iwasaki, J. M. Izen, V. Izzo, B. Jackson, M. Jackson, P. Jackson, M. R. Jaekel, V. Jain, K. Jakobs, S. Jakobsen, T. Jakoubek, J. Jakubek, D. O. Jamin, D. K. Jana, E. Jansen, H. Jansen, J. Janssen, M. Janus, G. Jarlskog, N. Javadov, T. Javůrek, L. Jeanty, G.-Y. Jeng, D. Jennens, P. Jenni, J. Jentzsch, C. Jeske, S. Jézéquel, H. Ji, W. Ji, J. Jia, Y. Jiang, M. Jimenez Belenguer, S. Jin, A. Jinaru, O. Jinnouchi, M. D. Joergensen, K. E. Johansson, P. Johansson, K. A. Johns, K. Jon-And, G. Jones, R. W. L. Jones, T. J. Jones, J. Jongmanns, P. M. Jorge, K. D. Joshi, J. Jovicevic, X. Ju, C. A. Jung, R. M. Jungst, P. Jussel, A. Juste Rozas, M. Kaci, A. Kaczmarska, M. Kado, H. Kagan, M. Kagan, E. Kajomovitz, S. Kama, N. Kanaya, M. Kaneda, S. Kaneti, T. Kanno, V. A. Kantserov, J. Kanzaki, B. Kaplan, A. Kapliy, D. Kar, K. Karakostas, N. Karastathis, M. Karnevskiy, S. N. Karpov, K. Karthik, V. Kartvelishvili, A. N. Karyukhin, L. Kashif, G. Kasieczka, R. D. Kass, A. Kastanas, Y. Kataoka, A. Katre, J. Katzy, V. Kaushik, K. Kawagoe, T. Kawamoto, G. Kawamura, S. Kazama, V. F. Kazanin, M. Y. Kazarinov, R. Keeler, R. Kehoe, M. Keil, J. S. Keller, H. Keoshkerian, O. Kepka, B. P. Kerševan, S. Kersten, K. Kessoku, J. Keung, F. Khalil-zada, H. Khandanyan, A. Khanov, A. Khodinov, A. Khomich, T. J. Khoo, G. Khoriauli, A. Khoroshilov, V. Khovanskiy, E. Khramov, J. Khubua, H. Y. Kim, H. Kim, S. H. Kim, N. Kimura, O. Kind, B. T. King, M. King, R. S. B. King, S. B. King, J. Kirk, A. E. Kiryunin, T. Kishimoto, D. Kisielewska, F. Kiss, T. Kitamura, T. Kittelmann, K. Kiuchi, E. Kladiva, M. Klein, U. Klein, K. Kleinknecht, P. Klimek, A. Klimentov, R. Klingenberg, J. A. Klinger, T. Klioutchnikova, P. F. Klok, E.-E. Kluge, P. Kluit, S. Kluth, E. Kneringer, E. B. F. G. Knoops, A. Knue, T. Kobayashi, M. Kobel, M. Kocian, P. Kodys, P. Koevesarki, T. Koffas, E. Koffeman, L. A. Kogan, S. Kohlmann, Z. Kohout, T. Kohriki, T. Koi, H. Kolanoski, I. Koletsou, J. Koll, A. A. Komar, Y. Komori, T. Kondo, N. Kondrashova, K. Köneke, A. C. König, S. König, T. Kono, R. Konoplich, N. Konstantinidis, R. Kopeliansky, S. Koperny, L. Köpke, A. K. Kopp, K. Korcyl, K. Kordas, A. Korn, A. A. Korol, I. Korolkov, E. V. Korolkova, V. A. Korotkov, O. Kortner, S. Kortner, V. V. Kostyukhin, V. M. Kotov, A. Kotwal, C. Kourkoumelis, V. Kouskoura, A. Koutsman, R. Kowalewski, T. Z. Kowalski, W. Kozanecki, A. S. Kozhin, V. Kral, V. A. Kramarenko, G. Kramberger, D. Krasnopevtsev, M. W. Krasny, A. Krasznahorkay, J. K. Kraus, A. Kravchenko, S. Kreiss, M. Kretz, J. Kretzschmar, K. Kreutzfeldt, P. Krieger, K. Kroeninger, H. Kroha, J. Kroll, J. Kroseberg, J. Krstic, U. Kruchonak, H. Krüger, T. Kruker, N. Krumnack, Z. V. Krumshteyn, A. Kruse, M. C. Kruse, M. Kruskal, T. Kubota, S. Kuday, S. Kuehn, A. Kugel, A. Kuhl, T. Kuhl, V. Kukhtin, Y. Kulchitsky, S. Kuleshov, M. Kuna, J. Kunkle, A. Kupco, H. Kurashige, Y. A. Kurochkin, R. Kurumida, V. Kus, E. S. Kuwertz, M. Kuze, J. Kvita, A. La Rosa, L. La Rotonda, C. Lacasta, F. Lacava, J. Lacey, H. Lacker, D. Lacour, V. R. Lacuesta, E. Ladygin, R. Lafaye, B. Laforge, T. Lagouri, S. Lai, H. Laier, L. Lambourne, S. Lammers, C. L. Lampen, W. Lampl, E. Lançon, U. Landgraf, M. P. J. Landon, V. S. Lang, C. Lange, A. J. Lankford, F. Lanni, K. Lantzsch, S. Laplace, C. Lapoire, J. F. Laporte, T. Lari, M. Lassnig, P. Laurelli, W. Lavrijsen, A. T. Law, P. Laycock, B. T. Le, O. Le Dortz, E. Le Guirriec, E. Le Menedeu, T. LeCompte, F. Ledroit-Guillon, C. A. Lee, H. Lee, J. S. H. Lee, S. C. Lee, L. Lee, G. Lefebvre, M. Lefebvre, F. Legger, C. Leggett, A. Lehan, M. Lehmacher, G. Lehmann Miotto, X. Lei, A. G. Leister, M. A. L. Leite, R. Leitner, D. Lellouch, B. Lemmer, K. J. C. Leney, T. Lenz, G. Lenzen, B. Lenzi, R. Leone, K. Leonhardt, S. Leontsinis, C. Leroy, C. G. Lester, C. M. Lester, M. Levchenko, J. Levêque, D. Levin, L. J. Levinson, M. Levy, A. Lewis, G. H. Lewis, A. M. Leyko, M. Leyton, B. Li, B. Li, H. Li, H. L. Li, L. Li, S. Li, Y. Li, Z. Liang, H. Liao, B. Liberti, P. Lichard, K. Lie, J. Liebal, W. Liebig, C. Limbach, A. Limosani, M. Limper, S. C. Lin, F. Linde, B. E. Lindquist, J. T. Linnemann, E. Lipeles, A. Lipniacka, M. Lisovyi, T. M. Liss, D. Lissauer, A. Lister, A. M. Litke, B. Liu, D. Liu, J. B. Liu, K. Liu, L. Liu, M. Liu, M. Liu, Y. Liu, M. Livan, S. S. A. Livermore, A. Lleres, J. Llorente Merino, S. L. Lloyd, F. Lo Sterzo, E. Lobodzinska, P. Loch, W. S. Lockman, T. Loddenkoetter, F. K. Loebinger, A. E. Loevschall-Jensen, A. Loginov, C. W. Loh, T. Lohse, K. Lohwasser, M. Lokajicek, V. P. Lombardo, B. A. Long, J. D. Long, R. E. Long, L. Lopes, D. Lopez Mateos, B. Lopez Paredes, J. Lorenz, N. Lorenzo Martinez, M. Losada, P. Loscutoff, X. Lou, A. Lounis, J. Love, P. A. Love, A. J. Lowe, F. Lu, H. J. Lubatti, C. Luci, A. Lucotte, F. Luehring, W. Lukas, L. Luminari, O. Lundberg, B. Lund-Jensen, M. Lungwitz, D. Lynn, R. Lysak, E. Lytken, H. Ma, L. L. Ma, G. Maccarrone, A. Macchiolo, J. Machado Miguens, D. Macina, D. Madaffari, R. Madar, H. J. Maddocks, W. F. Mader, A. Madsen, M. Maeno, T. Maeno, E. Magradze, K. Mahboubi, J. Mahlstedt, S. Mahmoud, C. Maiani, C. Maidantchik, A. Maio, S. Majewski, Y. Makida, N. Makovec, P. Mal, B. Malaescu, Pa. Malecki, V. P. Maleev, F. Malek, U. Mallik, D. Malon, C. Malone, S. Maltezos, V. M. Malyshev, S. Malyukov, J. Mamuzic, B. Mandelli, L. Mandelli, I. Mandić, R. Mandrysch, J. Maneira, A. Manfredini, L. Manhaes de Andrade Filho, J. A. Manjarres Ramos, A. Mann, P. M. Manning, A. Manousakis-Katsikakis, B. Mansoulie, R. Mantifel, L. Mapelli, L. March, J. F. Marchand, G. Marchiori, M. Marcisovsky, C. P. Marino, C. N. Marques, F. Marroquim, S. P. Marsden, Z. Marshall, L. F. Marti, S. Marti-Garcia, B. Martin, B. Martin, T. A. Martin, V. J. Martin, B. Martin dit Latour, H. Martinez, M. Martinez, S. Martin-Haugh, A. C. Martyniuk, M. Marx, F. Marzano, A. Marzin, L. Masetti, T. Mashimo, R. Mashinistov, J. Masik, A. L. Maslennikov, I. Massa, N. Massol, P. Mastrandrea, A. Mastroberardino, T. Masubuchi, H. Matsunaga, T. Matsushita, P. Mättig, S. Mättig, J. Mattmann, J. Maurer, S. J. Maxfield, D. A. Maximov, R. Mazini, L. Mazzaferro, G. Mc Goldrick, S. P. Mc Kee, A. McCarn, R. L. McCarthy, T. G. McCarthy, N. A. McCubbin, K. W. McFarlane, J. A. Mcfayden, G. Mchedlidze, T. Mclaughlan, S. J. McMahon, R. A. McPherson, A. Meade, J. Mechnich, M. Medinnis, S. Meehan, S. Mehlhase, A. Mehta, K. Meier, C. Meineck, B. Meirose, C. Melachrinos, B. R. Mellado Garcia, F. Meloni, A. Mengarelli, S. Menke, E. Meoni, K. M. Mercurio, S. Mergelmeyer, N. Meric, P. Mermod, L. Merola, C. Meroni, F. S. Merritt, H. Merritt, A. Messina, J. Metcalfe, A. S. Mete, C. Meyer, C. Meyer, J-P. Meyer, J. Meyer, R. P. Middleton, S. Migas, L. Mijović, G. Mikenberg, M. Mikestikova, M. Mikuž, D. W. Miller, C. Mills, A. Milov, D. A. Milstead, D. Milstein, A. A. Minaenko, I. A. Minashvili, A. I. Mincer, B. Mindur, M. Mineev, Y. Ming, L. M. Mir, G. Mirabelli, T. Mitani, J. Mitrevski, V. A. Mitsou, S. Mitsui, A. Miucci, P. S. Miyagawa, J. U. Mjörnmark, T. Moa, K. Mochizuki, V. Moeller, S. Mohapatra, W. Mohr, S. Molander, R. Moles-Valls, K. Mönig, C. Monini, J. Monk, E. Monnier, J. Montejo Berlingen, F. Monticelli, S. Monzani, R. W. Moore, A. Moraes, N. Morange, J. Morel, D. Moreno, M. Moreno Llácer, P. Morettini, M. Morgenstern, M. Morii, S. Moritz, A. K. Morley, G. Mornacchi, J. D. Morris, L. Morvaj, H. G. Moser, M. Mosidze, J. Moss, R. Mount, E. Mountricha, S. V. Mouraviev, E. J. W. Moyse, S. Muanza, R. D. Mudd, F. Mueller, J. Mueller, K. Mueller, T. Mueller, T. Mueller, D. Muenstermann, Y. Munwes, J. A. Murillo Quijada, W. J. Murray, H. Musheghyan, E. Musto, A. G. Myagkov, M. Myska, O. Nackenhorst, J. Nadal, K. Nagai, R. Nagai, Y. Nagai, K. Nagano, A. Nagarkar, Y. Nagasaka, M. Nagel, A. M. Nairz, Y. Nakahama, K. Nakamura, T. Nakamura, I. Nakano, H. Namasivayam, G. Nanava, R. Narayan, T. Nattermann, T. Naumann, G. Navarro, R. Nayyar, H. A. Neal, P. Yu. Nechaeva, T. J. Neep, A. Negri, G. Negri, M. Negrini, S. Nektarijevic, A. Nelson, T. K. Nelson, S. Nemecek, P. Nemethy, A. A. Nepomuceno, M. Nessi, M. S. Neubauer, M. Neumann, R. M. Neves, P. Nevski, P. R. Newman, D. H. Nguyen, R. B. Nickerson, R. Nicolaidou, B. Nicquevert, J. Nielsen, N. Nikiforou, A. Nikiforov, V. Nikolaenko, I. Nikolic-Audit, K. Nikolics, K. Nikolopoulos, P. Nilsson, Y. Ninomiya, A. Nisati, R. Nisius, T. Nobe, L. Nodulman, M. Nomachi, I. Nomidis, S. Norberg, M. Nordberg, S. Nowak, M. Nozaki, L. Nozka, K. Ntekas, G. Nunes Hanninger, T. Nunnemann, E. Nurse, F. Nuti, B. J. O’Brien, F. O’grady, D. C. O’Neil, V. O’Shea, F. G. Oakham, H. Oberlack, T. Obermann, J. Ocariz, A. Ochi, M. I. Ochoa, S. Oda, S. Odaka, H. Ogren, A. Oh, S. H. Oh, C. C. Ohm, H. Ohman, T. Ohshima, W. Okamura, H. Okawa, Y. Okumura, T. Okuyama, A. Olariu, A. G. Olchevski, S. A. Olivares Pino, D. Oliveira Damazio, E. Oliver Garcia, A. Olszewski, J. Olszowska, A. Onofre, P. U. E. Onyisi, C. J. Oram, M. J. Oreglia, Y. Oren, D. Orestano, N. Orlando, C. Oropeza Barrera, R. S. Orr, B. Osculati, R. Ospanov, G. Otero y Garzon, H. Otono, M. Ouchrif, E. A. Ouellette, F. Ould-Saada, A. Ouraou, K. P. Oussoren, Q. Ouyang, A. Ovcharova, M. Owen, V. E. Ozcan, N. Ozturk, K. Pachal, A. Pacheco Pages, C. Padilla Aranda, M. Pagáčová, S. Pagan Griso, E. Paganis, C. Pahl, F. Paige, P. Pais, K. Pajchel, G. Palacino, S. Palestini, D. Pallin, A. Palma, J. D. Palmer, Y. B. Pan, E. Panagiotopoulou, J. G. Panduro Vazquez, P. Pani, N. Panikashvili, S. Panitkin, D. Pantea, L. Paolozzi, Th. D. Papadopoulou, K. Papageorgiou, A. Paramonov, D. Paredes Hernandez, M. A. Parker, F. Parodi, J. A. Parsons, U. Parzefall, E. Pasqualucci, S. Passaggio, A. Passeri, F. Pastore, Fr. Pastore, G. Pásztor, S. Pataraia, N. D. Patel, J. R. Pater, S. Patricelli, T. Pauly, J. Pearce, M. Pedersen, S. Pedraza Lopez, R. Pedro, S. V. Peleganchuk, D. Pelikan, H. Peng, B. Penning, J. Penwell, D. V. Perepelitsa, E. Perez Codina, M. T. Pérez García-Estañ, V. Perez Reale, L. Perini, H. Pernegger, R. Perrino, R. Peschke, V. D. Peshekhonov, K. Peters, R. F. Y. Peters, B. A. Petersen, J. Petersen, T. C. Petersen, E. Petit, A. Petridis, C. Petridou, E. Petrolo, F. Petrucci, M. Petteni, N. E. Pettersson, R. Pezoa, P. W. Phillips, G. Piacquadio, E. Pianori, A. Picazio, E. Piccaro, M. Piccinini, R. Piegaia, D. T. Pignotti, J. E. Pilcher, A. D. Pilkington, J. Pina, M. Pinamonti, A. Pinder, J. L. Pinfold, A. Pingel, B. Pinto, S. Pires, M. Pitt, C. Pizio, M.-A. Pleier, V. Pleskot, E. Plotnikova, P. Plucinski, S. Poddar, F. Podlyski, R. Poettgen, L. Poggioli, D. Pohl, M. Pohl, G. Polesello, A. Policicchio, R. Polifka, A. Polini, C. S. Pollard, V. Polychronakos, K. Pommès, L. Pontecorvo, B. G. Pope, G. A. Popeneciu, D. S. Popovic, A. Poppleton, X. Portell Bueso, G. E. Pospelov, S. Pospisil, K. Potamianos, I. N. Potrap, C. J. Potter, C. T. Potter, G. Poulard, J. Poveda, V. Pozdnyakov, P. Pralavorio, A. Pranko, S. Prasad, R. Pravahan, S. Prell, D. Price, J. Price, L. E. Price, D. Prieur, M. Primavera, M. Proissl, K. Prokofiev, F. Prokoshin, E. Protopapadaki, S. Protopopescu, J. Proudfoot, M. Przybycien, H. Przysiezniak, E. Ptacek, E. Pueschel, D. Puldon, M. Purohit, P. Puzo, J. Qian, G. Qin, Y. Qin, A. Quadt, D. R. Quarrie, W. B. Quayle, D. Quilty, A. Qureshi, V. Radeka, V. Radescu, S. K. Radhakrishnan, P. Radloff, P. Rados, F. Ragusa, G. Rahal, S. Rajagopalan, M. Rammensee, A. S. Randle-Conde, C. Rangel-Smith, K. Rao, F. Rauscher, T. C. Rave, T. Ravenscroft, M. Raymond, A. L. Read, D. M. Rebuzzi, A. Redelbach, G. Redlinger, R. Reece, K. Reeves, L. Rehnisch, A. Reinsch, H. Reisin, M. Relich, C. Rembser, Z. L. Ren, A. Renaud, M. Rescigno, S. Resconi, O. L. Rezanova, P. Reznicek, R. Rezvani, R. Richter, M. Ridel, P. Rieck, M. Rijssenbeek, A. Rimoldi, L. Rinaldi, E. Ritsch, I. Riu, F. Rizatdinova, E. Rizvi, S. H. Robertson, A. Robichaud-Veronneau, D. Robinson, J. E. M. Robinson, A. Robson, C. Roda, L. Rodrigues, S. Roe, O. Røhne, S. Rolli, A. Romaniouk, M. Romano, G. Romeo, E. Romero Adam, N. Rompotis, L. Roos, E. Ros, S. Rosati, K. Rosbach, M. Rose, P. L. Rosendahl, O. Rosenthal, V. Rossetti, E. Rossi, L. P. Rossi, R. Rosten, M. Rotaru, I. Roth, J. Rothberg, D. Rousseau, C. R. Royon, A. Rozanov, Y. Rozen, X. Ruan, F. Rubbo, I. Rubinskiy, V. I. Rud, C. Rudolph, M. S. Rudolph, F. Rühr, A. Ruiz-Martinez, Z. Rurikova, N. A. Rusakovich, A. Ruschke, J. P. Rutherfoord, N. Ruthmann, Y. F. Ryabov, M. Rybar, G. Rybkin, N. C. Ryder, A. F. Saavedra, S. Sacerdoti, A. Saddique, I. Sadeh, H. F-W. Sadrozinski, R. Sadykov, F. Safai Tehrani, H. Sakamoto, Y. Sakurai, G. Salamanna, A. Salamon, M. Saleem, D. Salek, P. H. Sales De Bruin, D. Salihagic, A. Salnikov, J. Salt, B. M. Salvachua Ferrando, D. Salvatore, F. Salvatore, A. Salvucci, A. Salzburger, D. Sampsonidis, A. Sanchez, J. Sánchez, V. Sanchez Martinez, H. Sandaker, R. L. Sandbach, H. G. Sander, M. P. Sanders, M. Sandhoff, T. Sandoval, C. Sandoval, R. Sandstroem, D. P. C. Sankey, A. Sansoni, C. Santoni, R. Santonico, H. Santos, I. Santoyo Castillo, K. Sapp, A. Sapronov, J. G. Saraiva, B. Sarrazin, G. Sartisohn, O. Sasaki, Y. Sasaki, G. Sauvage, E. Sauvan, P. Savard, D. O. Savu, C. Sawyer, L. Sawyer, D. H. Saxon, J. Saxon, C. Sbarra, A. Sbrizzi, T. Scanlon, D. A. Scannicchio, M. Scarcella, J. Schaarschmidt, P. Schacht, D. Schaefer, R. Schaefer, S. Schaepe, S. Schaetzel, U. Schäfer, A. C. Schaffer, D. Schaile, R. D. Schamberger, V. Scharf, V. A. Schegelsky, D. Scheirich, M. Schernau, M. I. Scherzer, C. Schiavi, J. Schieck, C. Schillo, M. Schioppa, S. Schlenker, E. Schmidt, K. Schmieden, C. Schmitt, C. Schmitt, S. Schmitt, B. Schneider, Y. J. Schnellbach, U. Schnoor, L. Schoeffel, A. Schoening, B. D. Schoenrock, A. L. S. Schorlemmer, M. Schott, D. Schouten, J. Schovancova, S. Schramm, M. Schreyer, C. Schroeder, N. Schuh, M. J. Schultens, H.-C. Schultz-Coulon, H. Schulz, M. Schumacher, B. A. Schumm, Ph. Schune, A. Schwartzman, Ph. Schwegler, Ph. Schwemling, R. Schwienhorst, J. Schwindling, T. Schwindt, M. Schwoerer, F. G. Sciacca, E. Scifo, G. Sciolla, W. G. Scott, F. Scuri, F. Scutti, J. Searcy, G. Sedov, E. Sedykh, S. C. Seidel, A. Seiden, F. Seifert, J. M. Seixas, G. Sekhniaidze, S. J. Sekula, K. E. Selbach, D. M. Seliverstov, G. Sellers, N. Semprini-Cesari, C. Serfon, L. Serin, L. Serkin, T. Serre, R. Seuster, H. Severini, F. Sforza, A. Sfyrla, E. Shabalina, M. Shamim, L. Y. Shan, J. T. Shank, Q. T. Shao, M. Shapiro, P. B. Shatalov, K. Shaw, P. Sherwood, S. Shimizu, C. O. Shimmin, M. Shimojima, M. Shiyakova, A. Shmeleva, M. J. Shochet, D. Short, S. Shrestha, E. Shulga, M. A. Shupe, S. Shushkevich, P. Sicho, D. Sidorov, A. Sidoti, F. Siegert, Dj. Sijacki, O. Silbert, J. Silva, Y. Silver, D. Silverstein, S. B. Silverstein, V. Simak, O. Simard, Lj. Simic, S. Simion, E. Simioni, B. Simmons, R. Simoniello, M. Simonyan, P. Sinervo, N. B. Sinev, V. Sipica, G. Siragusa, A. Sircar, A. N. Sisakyan, S. Yu. Sivoklokov, J. Sjölin, T. B. Sjursen, H. P. Skottowe, K. Yu. Skovpen, P. Skubic, M. Slater, T. Slavicek, K. Sliwa, V. Smakhtin, B. H. Smart, L. Smestad, S. Yu. Smirnov, Y. Smirnov, L. N. Smirnova, O. Smirnova, K. M. Smith, M. Smizanska, K. Smolek, A. A. Snesarev, G. Snidero, S. Snyder, R. Sobie, F. Socher, A. Soffer, D. A. Soh, C. A. Solans, M. Solar, J. Solc, E. Yu. Soldatov, U. Soldevila, E. Solfaroli Camillocci, A. A. Solodkov, O. V. Solovyanov, V. Solovyev, P. Sommer, H. Y. Song, N. Soni, A. Sood, A. Sopczak, V. Sopko, B. Sopko, V. Sorin, M. Sosebee, R. Soualah, P. Soueid, A. M. Soukharev, D. South, S. Spagnolo, F. Spanò, W. R. Spearman, R. Spighi, G. Spigo, M. Spousta, T. Spreitzer, B. Spurlock, R. D. St. Denis, S. Staerz, J. Stahlman, R. Stamen, E. Stanecka, R. W. Stanek, C. Stanescu, M. Stanescu-Bellu, M. M. Stanitzki, S. Stapnes, E. A. Starchenko, J. Stark, P. Staroba, P. Starovoitov, R. Staszewski, P. Stavina, G. Steele, P. Steinberg, B. Stelzer, H. J. Stelzer, O. Stelzer-Chilton, H. Stenzel, S. Stern, G. A. Stewart, J. A. Stillings, M. C. Stockton, M. Stoebe, G. Stoicea, P. Stolte, S. Stonjek, A. R. Stradling, A. Straessner, M. E. Stramaglia, J. Strandberg, S. Strandberg, A. Strandlie, E. Strauss, M. Strauss, P. Strizenec, R. Ströhmer, D. M. Strom, R. Stroynowski, S. A. Stucci, B. Stugu, N. A. Styles, D. Su, J. Su, HS. Subramania, R. Subramaniam, A. Succurro, Y. Sugaya, C. Suhr, M. Suk, V. V. Sulin, S. Sultansoy, T. Sumida, X. Sun, J. E. Sundermann, K. Suruliz, G. Susinno, M. R. Sutton, Y. Suzuki, M. Svatos, S. Swedish, M. Swiatlowski, I. Sykora, T. Sykora, D. Ta, K. Tackmann, J. Taenzer, A. Taffard, R. Tafirout, N. Taiblum, Y. Takahashi, H. Takai, R. Takashima, H. Takeda, T. Takeshita, Y. Takubo, M. Talby, A. A. Talyshev, J. Y. C. Tam, M. C. Tamsett, K. G. Tan, J. Tanaka, R. Tanaka, S. Tanaka, S. Tanaka, A. J. Tanasijczuk, K. Tani, N. Tannoury, S. Tapprogge, S. Tarem, F. Tarrade, G. F. Tartarelli, P. Tas, M. Tasevsky, T. Tashiro, E. Tassi, A. Tavares Delgado, Y. Tayalati, F. E. Taylor, G. N. Taylor, W. Taylor, F. A. Teischinger, M. Teixeira Dias Castanheira, P. Teixeira-Dias, K. K. Temming, H. Ten Kate, P. K. Teng, S. Terada, K. Terashi, J. Terron, S. Terzo, M. Testa, R. J. Teuscher, J. Therhaag, T. Theveneaux-Pelzer, S. Thoma, J. P. Thomas, J. Thomas-Wilsker, E. N. Thompson, P. D. Thompson, P. D. Thompson, A. S. Thompson, L. A. Thomsen, E. Thomson, M. Thomson, W. M. Thong, R. P. Thun, F. Tian, M. J. Tibbetts, V. O. Tikhomirov, Yu. A. Tikhonov, S. Timoshenko, E. Tiouchichine, P. Tipton, S. Tisserant, T. Todorov, S. Todorova-Nova, B. Toggerson, J. Tojo, S. Tokár, K. Tokushuku, K. Tollefson, L. Tomlinson, M. Tomoto, L. Tompkins, K. Toms, N. D. Topilin, E. Torrence, H. Torres, E. Torró Pastor, J. Toth, F. Touchard, D. R. Tovey, H. L. Tran, T. Trefzger, L. Tremblet, A. Tricoli, I. M. Trigger, S. Trincaz-Duvoid, M. F. Tripiana, N. Triplett, W. Trischuk, B. Trocmé, C. Troncon, M. Trottier-McDonald, M. Trovatelli, P. True, M. Trzebinski, A. Trzupek, C. Tsarouchas, J. C-L. Tseng, P. V. Tsiareshka, D. Tsionou, G. Tsipolitis, N. Tsirintanis, S. Tsiskaridze, V. Tsiskaridze, E. G. Tskhadadze, I. I. Tsukerman, V. Tsulaia, S. Tsuno, D. Tsybychev, A. Tudorache, V. Tudorache, A. N. Tuna, S. A. Tupputi, S. Turchikhin, D. Turecek, I. Turk Cakir, R. Turra, P. M. Tuts, A. Tykhonov, M. Tylmad, M. Tyndel, K. Uchida, I. Ueda, R. Ueno, M. Ughetto, M. Ugland, M. Uhlenbrock, F. Ukegawa, G. Unal, A. Undrus, G. Unel, F. C. Ungaro, Y. Unno, D. Urbaniec, P. Urquijo, G. Usai, A. Usanova, L. Vacavant, V. Vacek, B. Vachon, N. Valencic, S. Valentinetti, A. Valero, L. Valery, S. Valkar, E. Valladolid Gallego, S. Vallecorsa, J. A. Valls Ferrer, P. C. Van Der Deijl, R. van der Geer, H. van der Graaf, R. Van Der Leeuw, D. van der Ster, N. van Eldik, P. van Gemmeren, J. Van Nieuwkoop, I. van Vulpen, M. C. van Woerden, M. Vanadia, W. Vandelli, R. Vanguri, A. Vaniachine, P. Vankov, F. Vannucci, G. Vardanyan, R. Vari, E. W. Varnes, T. Varol, D. Varouchas, A. Vartapetian, K. E. Varvell, F. Vazeille, T. Vazquez Schroeder, J. Veatch, F. Veloso, S. Veneziano, A. Ventura, D. Ventura, M. Venturi, N. Venturi, A. Venturini, V. Vercesi, M. Verducci, W. Verkerke, J. C. Vermeulen, A. Vest, M. C. Vetterli, O. Viazlo, I. Vichou, T. Vickey, O. E. Vickey Boeriu, G. H. A. Viehhauser, S. Viel, R. Vigne, M. Villa, M. Villaplana Perez, E. Vilucchi, M. G. Vincter, V. B. Vinogradov, J. Virzi, I. Vivarelli, F. Vives Vaque, S. Vlachos, D. Vladoiu, M. Vlasak, A. Vogel, P. Vokac, G. Volpi, M. Volpi, H. von der Schmitt, H. von Radziewski, E. von Toerne, V. Vorobel, K. Vorobev, M. Vos, R. Voss, J. H. Vossebeld, N. Vranjes, M. Vranjes Milosavljevic, V. Vrba, M. Vreeswijk, T. Vu Anh, R. Vuillermet, I. Vukotic, Z. Vykydal, W. Wagner, P. Wagner, S. Wahrmund, J. Wakabayashi, J. Walder, R. Walker, W. Walkowiak, R. Wall, P. Waller, B. Walsh, C. Wang, C. Wang, F. Wang, H. Wang, H. Wang, J. Wang, J. Wang, K. Wang, R. Wang, S. M. Wang, T. Wang, X. Wang, C. Wanotayaroj, A. Warburton, C. P. Ward, D. R. Wardrope, M. Warsinsky, A. Washbrook, C. Wasicki, I. Watanabe, P. M. Watkins, A. T. Watson, I. J. Watson, M. F. Watson, G. Watts, S. Watts, B. M. Waugh, S. Webb, M. S. Weber, S. W. Weber, J. S. Webster, A. R. Weidberg, P. Weigell, B. Weinert, J. Weingarten, C. Weiser, H. Weits, P. S. Wells, T. Wenaus, D. Wendland, Z. Weng, T. Wengler, S. Wenig, N. Wermes, M. Werner, P. Werner, M. Wessels, J. Wetter, K. Whalen, A. White, M. J. White, R. White, S. White, D. Whiteson, D. Wicke, F. J. Wickens, W. Wiedenmann, M. Wielers, P. Wienemann, C. Wiglesworth, L. A. M. Wiik-Fuchs, P. A. Wijeratne, A. Wildauer, M. A. Wildt, H. G. Wilkens, J. Z. Will, H. H. Williams, S. Williams, C. Willis, S. Willocq, J. A. Wilson, A. Wilson, I. Wingerter-Seez, F. Winklmeier, M. Wittgen, T. Wittig, J. Wittkowski, S. J. Wollstadt, M. W. Wolter, H. Wolters, B. K. Wosiek, J. Wotschack, M. J. Woudstra, K. W. Wozniak, M. Wright, M. Wu, S. L. Wu, X. Wu, Y. Wu, E. Wulf, T. R. Wyatt, B. M. Wynne, S. Xella, M. Xiao, D. Xu, L. Xu, B. Yabsley, S. Yacoob, M. Yamada, H. Yamaguchi, Y. Yamaguchi, A. Yamamoto, K. Yamamoto, S. Yamamoto, T. Yamamura, T. Yamanaka, K. Yamauchi, Y. Yamazaki, Z. Yan, H. Yang, H. Yang, U. K. Yang, Y. Yang, S. Yanush, L. Yao, W-M. Yao, Y. Yasu, E. Yatsenko, K. H. Yau Wong, J. Ye, S. Ye, A. L. Yen, E. Yildirim, M. Yilmaz, R. Yoosoofmiya, K. Yorita, R. Yoshida, K. Yoshihara, C. Young, C. J. S. Young, S. Youssef, D. R. Yu, J. Yu, J. M. Yu, J. Yu, L. Yuan, A. Yurkewicz, B. Zabinski, R. Zaidan, A. M. Zaitsev, A. Zaman, S. Zambito, L. Zanello, D. Zanzi, A. Zaytsev, C. Zeitnitz, M. Zeman, A. Zemla, K. Zengel, O. Zenin, T. Ženiš, D. Zerwas, G. Zevi della Porta, D. Zhang, F. Zhang, H. Zhang, J. Zhang, L. Zhang, X. Zhang, Z. Zhang, Z. Zhao, A. Zhemchugov, J. Zhong, B. Zhou, L. Zhou, N. Zhou, C. G. Zhu, H. Zhu, J. Zhu, Y. Zhu, X. Zhuang, A. Zibell, D. Zieminska, N. I. Zimine, C. Zimmermann, R. Zimmermann, S. Zimmermann, S. Zimmermann, Z. Zinonos, M. Ziolkowski, G. Zobernig, A. Zoccoli, M. zur Nedden, G. Zurzolo, V. Zutshi, L. Zwalinski

**Affiliations:** 1 Department of Physics, University of Adelaide, Adelaide, Australia; 2 Physics Department, SUNY Albany, Albany, NY USA; 3 Department of Physics, University of Alberta, Edmonton, AB Canada; 4 Department of Physics, Ankara University, Ankara, Turkey; 200 Department of Physics, Gazi University, Ankara, Turkey; 201 Division of Physics, TOBB University of Economics and Technology, Ankara, Turkey; 202 Turkish Atomic Energy Authority, Ankara, Turkey; 5 LAPP, CNRS/IN2P3 and Université de Savoie, Annecy-le-Vieux, France; 6 High Energy Physics Division, Argonne National Laboratory, Argonne, IL USA; 7 Department of Physics, University of Arizona, Tucson, AZ USA; 8 Department of Physics, The University of Texas at Arlington, Arlington, TX USA; 9 Physics Department, University of Athens, Athens, Greece; 10 Physics Department, National Technical University of Athens, Zografou, Greece; 11 Institute of Physics, Azerbaijan Academy of Sciences, Baku, Azerbaijan; 12 Institut de Física d’Altes Energies and Departament de Física de la Universitat Autònoma de Barcelona, Barcelona, Spain; 13 Institute of Physics, University of Belgrade, Belgrade, Serbia; 203 Vinca Institute of Nuclear Sciences, University of Belgrade, Belgrade, Serbia; 14 Department for Physics and Technology, University of Bergen, Bergen, Norway; 15 Physics Division, Lawrence Berkeley National Laboratory and University of California, Berkeley, CA USA; 16 Department of Physics, Humboldt University, Berlin, Germany; 17 Albert Einstein Center for Fundamental Physics and Laboratory for High Energy Physics, University of Bern, Bern, Switzerland; 18 School of Physics and Astronomy, University of Birmingham, Birmingham, UK; 19 Department of Physics, Bogazici University, Istanbul, Turkey; 204 Department of Physics, Dogus University, Istanbul, Turkey; 205 Department of Physics Engineering, Gaziantep University, Gaziantep, Turkey; 20 INFN Sezione di Bologna, Bologna, Italy; 206 Dipartimento di Fisica e Astronomia, Università di Bologna, Bologna, Italy; 21 Physikalisches Institut, University of Bonn, Bonn, Germany; 22 Department of Physics, Boston University, Boston, MA USA; 23 Department of Physics, Brandeis University, Waltham, MA USA; 24 Universidade Federal do Rio De Janeiro COPPE/EE/IF, Rio de Janeiro, Brazil; 207 Federal University of Juiz de Fora (UFJF), Juiz de Fora, Brazil; 208 Federal University of Sao Joao del Rei (UFSJ), Sao Joao del Rei, Brazil; 209 Instituto de Fisica, Universidade de Sao Paulo, Sao Paulo, Brazil; 25 Physics Department, Brookhaven National Laboratory, Upton, NY USA; 26 National Institute of Physics and Nuclear Engineering, Bucharest, Romania; 210 Physics Department, National Institute for Research and Development of Isotopic and Molecular Technologies, Cluj Napoca, Romania; 211 University Politehnica Bucharest, Bucharest, Romania; 212 West University in Timisoara, Timisoara, Romania; 27 Departamento de Física, Universidad de Buenos Aires, Buenos Aires, Argentina; 28 Cavendish Laboratory, University of Cambridge, Cambridge, UK; 29 Department of Physics, Carleton University, Ottawa, ON Canada; 30 CERN, Geneva, Switzerland; 31 Enrico Fermi Institute, University of Chicago, Chicago, IL USA; 32 Departamento de Física, Pontificia Universidad Católica de Chile, Santiago, Chile; 213 Departamento de Física, Universidad Técnica Federico Santa María, Valparaiso, Chile; 33 Institute of High Energy Physics, Chinese Academy of Sciences, Beijing, China; 214 Department of Modern Physics, University of Science and Technology of China, Hefei, Anhui China; 215 Department of Physics, Nanjing University, Nanjing, Jiangsu China; 216 School of Physics, Shandong University, Jinan, Shandong China; 217 Physics Department, Shanghai Jiao Tong University, Shanghai, China; 34 Laboratoire de Physique Corpusculaire, Clermont Université and Université Blaise Pascal and CNRS/IN2P3, Clermont-Ferrand, France; 35 Nevis Laboratory, Columbia University, Irvington, NY USA; 36 Niels Bohr Institute, University of Copenhagen, Copenhagen, Denmark; 37 INFN Gruppo Collegato di Cosenza, Laboratori Nazionali di Frascati, Frascati, Italy; 218 Dipartimento di Fisica, Università della Calabria, Rende, Italy; 38 Faculty of Physics and Applied Computer Science, AGH University of Science and Technology, Kraków, Poland; 219 Marian Smoluchowski Institute of Physics, Jagiellonian University, Kraków, Poland; 39 The Henryk Niewodniczanski Institute of Nuclear Physics, Polish Academy of Sciences, Kraków, Poland; 40 Physics Department, Southern Methodist University, Dallas, TX USA; 41 Physics Department, University of Texas at Dallas, Richardson, TX USA; 42 DESY, Hamburg and Zeuthen, Germany; 43 Institut für Experimentelle Physik IV, Technische Universität Dortmund, Dortmund, Germany; 44 Institut für Kern- und Teilchenphysik, Technische Universität Dresden, Dresden, Germany; 45 Department of Physics, Duke University, Durham, NC USA; 46 SUPA-School of Physics and Astronomy, University of Edinburgh, Edinburgh, UK; 47 INFN Laboratori Nazionali di Frascati, Frascati, Italy; 48 Fakultät für Mathematik und Physik, Albert-Ludwigs-Universität, Freiburg, Germany; 49 Section de Physique, Université de Genève, Geneva, Switzerland; 50 INFN Sezione di Genova, Genoa, Italy; 220 Dipartimento di Fisica, Università di Genova, Genoa, Italy; 51 E. Andronikashvili Institute of Physics, Iv. Javakhishvili Tbilisi State University, Tbilisi, Georgia; 221 High Energy Physics Institute, Tbilisi State University, Tbilisi, Georgia; 52 II Physikalisches Institut, Justus-Liebig-Universität Giessen, Giessen, Germany; 53 SUPA-School of Physics and Astronomy, University of Glasgow, Glasgow, UK; 54 II Physikalisches Institut, Georg-August-Universität, Göttingen, Germany; 55 Laboratoire de Physique Subatomique et de Cosmologie, Université Grenoble-Alpes, CNRS/IN2P3, Grenoble, France; 56 Department of Physics, Hampton University, Hampton, VA USA; 57 Laboratory for Particle Physics and Cosmology, Harvard University, Cambridge, MA USA; 58 Kirchhoff-Institut für Physik, Ruprecht-Karls-Universität Heidelberg, Heidelberg, Germany; 222 Physikalisches Institut, Ruprecht-Karls-Universität Heidelberg, Heidelberg, Germany; 223 ZITI Institut für technische Informatik, Ruprecht-Karls-Universität Heidelberg, Mannheim, Germany; 59 Faculty of Applied Information Science, Hiroshima Institute of Technology, Hiroshima, Japan; 60 Department of Physics, Indiana University, Bloomington, IN USA; 61 Institut für Astro- und Teilchenphysik, Leopold-Franzens-Universität, Innsbruck, Austria; 62 University of Iowa, Iowa City, IA USA; 63 Department of Physics and Astronomy, Iowa State University, Ames, IA USA; 64 Joint Institute for Nuclear Research, JINR Dubna, Dubna, Russia; 65 KEK, High Energy Accelerator Research Organization, Tsukuba, Japan; 66 Graduate School of Science, Kobe University, Kobe, Japan; 67 Faculty of Science, Kyoto University, Kyoto, Japan; 68 Kyoto University of Education, Kyoto, Japan; 69 Department of Physics, Kyushu University, Fukuoka, Japan; 70 Instituto de Física La Plata, Universidad Nacional de La Plata and CONICET, La Plata, Argentina; 71 Physics Department, Lancaster University, Lancaster, UK; 72 INFN Sezione di Lecce, Lecce, Italy; 224 Dipartimento di Matematica e Fisica, Università del Salento, Lecce, Italy; 73 Oliver Lodge Laboratory, University of Liverpool, Liverpool, UK; 74 Department of Physics, Jožef Stefan Institute and University of Ljubljana, Ljubljana, Slovenia; 75 School of Physics and Astronomy, Queen Mary University of London, London, UK; 76 Department of Physics, Royal Holloway University of London, Surrey, UK; 77 Department of Physics and Astronomy, University College London, London, UK; 78 Louisiana Tech University, Ruston, LA USA; 79 Laboratoire de Physique Nucléaire et de Hautes Energies, UPMC and Université Paris-Diderot and CNRS/IN2P3, Paris, France; 80 Fysiska institutionen, Lunds universitet, Lund, Sweden; 81 Departamento de Fisica Teorica C-15, Universidad Autonoma de Madrid, Madrid, Spain; 82 Institut für Physik, Universität Mainz, Mainz, Germany; 83 School of Physics and Astronomy, University of Manchester, Manchester, UK; 84 CPPM, Aix-Marseille Université and CNRS/IN2P3, Marseille, France; 85 Department of Physics, University of Massachusetts, Amherst, MA USA; 86 Department of Physics, McGill University, Montreal, QC Canada; 87 School of Physics, University of Melbourne, Parkville, VIC Australia; 88 Department of Physics, The University of Michigan, Ann Arbor, MI USA; 89 Department of Physics and Astronomy, Michigan State University, East Lansing, MI USA; 90 INFN Sezione di Milano, Milan, Italy; 225 Dipartimento di Fisica, Università di Milano, Milan, Italy; 91 B.I. Stepanov Institute of Physics, National Academy of Sciences of Belarus, Minsk, Republic of Belarus; 92 National Scientific and Educational Centre for Particle and High Energy Physics, Minsk, Republic of Belarus; 93 Department of Physics, Massachusetts Institute of Technology, Cambridge, MA USA; 94 Group of Particle Physics, University of Montreal, Montreal, QC Canada; 95 P.N. Lebedev Institute of Physics, Academy of Sciences, Moscow, Russia; 96 Institute for Theoretical and Experimental Physics (ITEP), Moscow, Russia; 97 Moscow Engineering and Physics Institute (MEPhI), Moscow, Russia; 98 D.V.Skobeltsyn Institute of Nuclear Physics, M.V.Lomonosov Moscow State University, Moscow, Russia; 99 Fakultät für Physik, Ludwig-Maximilians-Universität München, Munich, Germany; 100 Max-Planck-Institut für Physik (Werner-Heisenberg-Institut), Munich, Germany; 101 Nagasaki Institute of Applied Science, Nagasaki, Japan; 102 Graduate School of Science and Kobayashi-Maskawa Institute, Nagoya University, Nagoya, Japan; 103 INFN Sezione di Napoli, Naples, Italy; 226 Dipartimento di Fisica, Università di Napoli, Naples, Italy; 104 Department of Physics and Astronomy, University of New Mexico, Albuquerque, NM USA; 105 Institute for Mathematics, Astrophysics and Particle Physics, Radboud University Nijmegen/Nikhef, Nijmegen, Netherlands; 106 Nikhef National Institute for Subatomic Physics and University of Amsterdam, Amsterdam, The Netherlands; 107 Department of Physics, Northern Illinois University, DeKalb, IL USA; 108 Budker Institute of Nuclear Physics, SB RAS, Novosibirsk, Russia; 109 Department of Physics, New York University, New York, NY USA; 110 Ohio State University, Columbus, OH USA; 111 Faculty of Science, Okayama University, Okayama, Japan; 112 Homer L. Dodge Department of Physics and Astronomy, University of Oklahoma, Norman, OK USA; 113 Department of Physics, Oklahoma State University, Stillwater, OK USA; 114 Palacký University, RCPTM, Olomouc, Czech Republic; 115 Center for High Energy Physics, University of Oregon, Eugene, OR USA; 116 LAL, Université Paris-Sud and CNRS/IN2P3, Orsay, France; 117 Graduate School of Science, Osaka University, Osaka, Japan; 118 Department of Physics, University of Oslo, Oslo, Norway; 119 Department of Physics, Oxford University, Oxford, UK; 120 INFN Sezione di Pavia, Pavia, Italy; 227 Dipartimento di Fisica, Università di Pavia, Pavia, Italy; 121 Department of Physics, University of Pennsylvania, Philadelphia, PA USA; 122 Petersburg Nuclear Physics Institute, Gatchina, Russia; 123 INFN Sezione di Pisa, Pisa, Italy; 228 Dipartimento di Fisica E. Fermi, Università di Pisa, Pisa, Italy; 124 Department of Physics and Astronomy, University of Pittsburgh, Pittsburgh, PA USA; 125 Laboratorio de Instrumentacao e Fisica Experimental de Particulas-LIP, Lisbon, Portugal; 229 Faculdade de Ciências, Universidade de Lisboa, Lisbon, Portugal; 230 Department of Physics, University of Coimbra, Coimbra, Portugal; 231 Centro de Física Nuclear da Universidade de Lisboa, Lisbon, Portugal; 232 Departamento de Fisica, Universidade do Minho, Braga, Portugal; 233 Departamento de Fisica Teorica y del Cosmos and CAFPE, Universidad de Granada, Granada, Spain; 234 Dep Fisica and CEFITEC of Faculdade de Ciencias e Tecnologia, Universidade Nova de Lisboa, Caparica, Portugal; 126 Institute of Physics, Academy of Sciences of the Czech Republic, Prague, Czech Republic; 127 Czech Technical University in Prague, Prague, Czech Republic; 128 Faculty of Mathematics and Physics, Charles University in Prague, Prague, Czech Republic; 129 State Research Center Institute for High Energy Physics, Protvino, Russia; 130 Particle Physics Department, Rutherford Appleton Laboratory, Didcot, UK; 131 Physics Department, University of Regina, Regina, SK Canada; 132 Ritsumeikan University, Kusatsu, Shiga Japan; 133 INFN Sezione di Roma, Rome, Italy; 235 Dipartimento di Fisica, Sapienza Università di Roma, Rome, Italy; 134 INFN Sezione di Roma Tor Vergata, Rome, Italy; 236 Dipartimento di Fisica, Università di Roma Tor Vergata, Rome, Italy; 135 INFN Sezione di Roma Tre, Rome, Italy; 237 Dipartimento di Matematica e Fisica, Università Roma Tre, Rome, Italy; 136 Faculté des Sciences Ain Chock, Réseau Universitaire de Physique des Hautes Energies-Université Hassan II, Casablanca, Morocco; 238 Centre National de l’Energie des Sciences Techniques Nucleaires, Rabat, Morocco; 239 Faculté des Sciences Semlalia, Université Cadi Ayyad, LPHEA-Marrakech, Marrakech, Morocco; 240 Faculté des Sciences, Université Mohamed Premier and LPTPM, Oujda, Morocco; 241 Faculté des Sciences, Université Mohammed V-Agdal, Rabat, Morocco; 137 DSM/IRFU (Institut de Recherches sur les Lois Fondamentales de l’Univers), CEA Saclay (Commissariat à l’Energie Atomique et aux Energies Alternatives), Gif-sur-Yvette, France; 138 Santa Cruz Institute for Particle Physics, University of California Santa Cruz, Santa Cruz, CA USA; 139 Department of Physics, University of Washington, Seattle, WA USA; 140 Department of Physics and Astronomy, University of Sheffield, Sheffield, UK; 141 Department of Physics, Shinshu University, Nagano, Japan; 142 Fachbereich Physik, Universität Siegen, Siegen, Germany; 143 Department of Physics, Simon Fraser University, Burnaby, BC Canada; 144 SLAC National Accelerator Laboratory, Stanford, CA USA; 145 Faculty of Mathematics, Physics and Informatics, Comenius University, Bratislava, Slovak Republic; 242 Department of Subnuclear Physics, Institute of Experimental Physics of the Slovak Academy of Sciences, Kosice, Slovak Republic; 146 Department of Physics, University of Cape Town, Cape Town, South Africa; 243 Department of Physics, University of Johannesburg, Johannesburg, South Africa; 244 School of Physics, University of the Witwatersrand, Johannesburg, South Africa; 147 Department of Physics, Stockholm University, Stockholm, Sweden; The Oskar Klein Centre, Stockholm, Sweden; 245 The Oskar Klein Centre, Stockholm, Sweden; 148 Physics Department, Royal Institute of Technology, Stockholm, Sweden; 149 Departments of Physics and Astronomy and Chemistry, Stony Brook University, Stony Brook, NY USA; 150 Department of Physics and Astronomy, University of Sussex, Brighton, UK; 151 School of Physics, University of Sydney, Sydney, Australia; 152 Institute of Physics, Academia Sinica, Taipei, Taiwan; 153 Department of Physics, Technion: Israel Institute of Technology, Haifa, Israel; 154 Raymond and Beverly Sackler School of Physics and Astronomy, Tel Aviv University, Tel Aviv, Israel; 155 Department of Physics, Aristotle University of Thessaloniki, Thessaloniki, Greece; 156 International Center for Elementary Particle Physics and Department of Physics, The University of Tokyo, Tokyo, Japan; 157 Graduate School of Science and Technology, Tokyo Metropolitan University, Tokyo, Japan; 158 Department of Physics, Tokyo Institute of Technology, Tokyo, Japan; 159 Department of Physics, University of Toronto, Toronto, ON Canada; 160 TRIUMF, Vancouver, BC Canada; 246 Department of Physics and Astronomy, York University, Toronto, ON Canada; 161 Faculty of Pure and Applied Sciences, University of Tsukuba, Tsukuba, Japan; 162 Department of Physics and Astronomy, Tufts University, Medford, MA USA; 163 Centro de Investigaciones, Universidad Antonio Narino, Bogota, Colombia; 164 Department of Physics and Astronomy, University of California Irvine, Irvine, CA USA; 165 INFN Gruppo Collegato di Udine, Sezione di Trieste, Udine, Italy; 247 ICTP, Trieste, Italy; 248 Dipartimento di Chimica, Fisica e Ambiente, Università di Udine, Udine, Italy; 166 Department of Physics, University of Illinois, Urbana, IL USA; 167 Department of Physics and Astronomy, University of Uppsala, Uppsala, Sweden; 168 Instituto de Física Corpuscular (IFIC) and Departamento de Física Atómica, Molecular y Nuclear and Departamento de Ingeniería Electrónica and Instituto de Microelectrónica de Barcelona (IMB-CNM), University of Valencia and CSIC, Valencia, Spain; 169 Department of Physics, University of British Columbia, Vancouver, BC Canada; 170 Department of Physics and Astronomy, University of Victoria, Victoria, BC Canada; 171 Department of Physics, University of Warwick, Coventry, UK; 172 Waseda University, Tokyo, Japan; 173 Department of Particle Physics, The Weizmann Institute of Science, Rehovot, Israel; 174 Department of Physics, University of Wisconsin, Madison, WI USA; 175 Fakultät für Physik und Astronomie, Julius-Maximilians-Universität, Würzburg, Germany; 176 Fachbereich C Physik, Bergische Universität Wuppertal, Wuppertal, Germany; 177 Department of Physics, Yale University, New Haven, CT USA; 178 Yerevan Physics Institute, Yerevan, Armenia; 179 Centre de Calcul de l’Institut National de Physique Nucléaire et de Physique des Particules (IN2P3), Villeurbanne, France; 180 CERN, 1211 Geneva 23, Switzerland

## Abstract

The integrated elliptic flow of charged particles produced in Pb+Pb collisions at $\sqrt{{s}_{\mathrm {NN}}}=2.76$ TeV has been measured with the ATLAS detector using data collected at the Large Hadron Collider. The anisotropy parameter, $v_2$, was measured in the pseudorapidity range $|\eta |\le 2.5$ with the event-plane method. In order to include tracks with very low transverse momentum $p_{\mathrm {T}}$, thus reducing the uncertainty in $v_2$ integrated over $p_{\mathrm {T}}$, a $1~\mu \hbox {b}^{-1}$ data sample recorded without a magnetic field in the tracking detectors is used. The centrality dependence of the integrated $v_2$ is compared to other measurements obtained with higher $p_{\mathrm {T}}$ thresholds. The integrated elliptic flow is weakly decreasing with $|\eta |$. The integrated $v_2$ transformed to the rest frame of one of the colliding nuclei is compared to the lower-energy RHIC data.

## Introduction

The anisotropy in the azimuthal angle distribution of particles produced in heavy-ion collisions has been studied extensively due to its sensitivity to the properties of the produced hadronic medium [1, 2]. The final-state anisotropy arises from the initial spatial asymmetry of the overlap zone in the collision of two nuclei with non-zero impact parameter. The initial spatial asymmetry induces asymmetric pressure gradients that are stronger in the direction of the reaction plane and, due to the collective expansion, lead to an azimuthally asymmetric distribution of the ejected particles. The final-state anisotropy is customarily characterized by the coefficients $v_n$ of the Fourier decomposition of the azimuthal angle distribution of the emitted particles [3]. The second Fourier coefficient $v_2$ is related to the elliptical shape of the overlap region in non-central heavy-ion collisions, and the higher flow harmonics reflect fluctuations in the initial collision geometry [4]. The first observation of elliptic flow, quantified by measurements of $v_2$, at RHIC [5–8] were found to be well described by predictions based on relativistic hydrodynamics [9–11], providing compelling evidence that the created matter is strongly coupled and behaves like an almost perfect, non-viscous, fluid. Later studies show small deviations from ideal hydrodynamics, described in terms of the ratio of shear viscosity to entropy density [12–15].

First results from Pb+Pb collisions at $\sqrt{s_{\mathrm {NN}}}=2.76$ TeV  [16–21] from the Large Hadron Collider (LHC) showed no change in the transverse momentum, $p_{\mathrm {T}}$, dependence of elliptic flow from that measured at the highest RHIC energy, while the elliptic flow integrated over $p_{\mathrm {T}}$ [16, 20] was found to increase by about 30 % from the RHIC energy of $\sqrt{s_{\mathrm {NN}}}=200$ GeV 1 to $\sqrt{s_{\mathrm {NN}}}=2.76$ TeV at the LHC. This increase in the integrated elliptic flow with energy is therefore driven mostly by the increase in the mean $p_{\mathrm {T}}$ of the produced particles. The dependence of elliptic flow on the geometry of the collision (the collision centrality) is of particular importance, since the flow is thought to depend strongly on the initial spatial anisotropy. Hydrodynamical models are used to quantitatively relate the initial geometry to the experimentally measured distributions. Furthermore, recent hydrodynamical calculations [22, 23] also include a longitudinal dependence in the source shape, which can be deduced from flow measurements over a wide pseudorapidity range.

This article presents measurements of the centrality and pseudorapidity dependence of the elliptic flow integrated over the $p_{\mathrm {T}}$ of charged particles produced in Pb+Pb collisions at $\sqrt{s_{\mathrm {NN}}}=2.76$ TeV recorded in 2010 by the ATLAS detector.

In order to reduce the uncertainty in the $p_{\mathrm {T}}$-integrated coefficient $v_2$ by including tracks with $p_{\mathrm {T}}$ lower than in the measurements reported by the ALICE [16] and CMS [20] experiments, a special track reconstruction procedure was applied to “field-off” data taken without the solenoid’s magnetic field in the tracking detectors. Other track reconstruction methods, applicable at higher $p_{\mathrm {T}}$, were exploited in cross-checks using “field-on” data taken with the solenoid’s magnetic field.

## The ATLAS detector

The ATLAS detector is a multi-purpose particle physics apparatus and is described in detail elsewhere [24]. This analysis uses the three-level trigger system to select Pb+Pb collision events, the forward calorimeters (FCal) to measure the collision centrality, and the inner detector (ID) to measure charged-particle tracks. The ID tracking system comprises silicon pixel and microstrip detectors and a transition radiation tracker. It provides complete azimuthal coverage and spans the pseudorapidity region $|\eta | < 2.5$.^2^ The pixel detector consists of a three-layer barrel section and three discs in each of the forward regions. The semiconductor tracker has four double layers of microstrip sensors in its barrel section and nine discs in each of the forward regions. The ID is surrounded by a thin superconducting solenoid, which produces a 2 T axial magnetic field for the field-on data. The FCal measures both electromagnetic and hadronic energy, using copper–tungsten/liquid-argon technology, and provides complete azimuthal coverage for $3.2<|\eta |<4.9$. The hardware-based Level-1 trigger selected minimum-bias Pb+Pb collisions by requiring either a coincidence of signals recorded in the zero-degree calorimeters (ZDC) located symmetrically at $z=\pm 140$ m ($|\eta |>8.3)$ or a signal in at least one side of the minimum-bias trigger scintillators (MBTS) at $z=\pm 3.6$ m ($2.1<|\eta |<3.9$). To suppress beam backgrounds, the Level-2 trigger demanded MBTS signals from opposite sides of the interaction region and imposed a timing requirement on them.

With these trigger conditions, ATLAS recorded a sample of Pb+Pb collisions corresponding to an integrated luminosity of approximately $1~\mu \hbox {b}^{-1}$ taken with the field provided by the solenoid turned off. In addition, approximately $0.5~\mu \hbox {b}^{-1}$ of field-on data was used in studies of track reconstruction performance.

## Event selection and centrality definition

The offline event selection required each event to have a vertex formed by at least three charged-particle tracks reconstructed in the ID. The data were recorded at low instantaneous luminosity where the probability of multiple collisions per bunch crossing (pile-up) was negligible. The track reconstruction algorithms therefore allowed only one collision vertex (called the primary vertex) in each event, thereby reducing the processing time while maintaining efficiency. The time difference between the MBTS signals from the opposite sides of the interaction region was required to be less than 3 ns, and a coincidence of ZDC signals was also required. These additional selection criteria efficiently remove beam-gas and photo-nuclear interactions. As shown in previous studies [18], the applied trigger and offline requirements provide a minimum-bias event sample in which the fraction of inelastic Pb+Pb collisions is $98\pm 2$ %.

Events satisfying the above criteria were also required to have a primary vertex within 50 mm (100 mm) in the z-direction of the nominal centre of the ATLAS detector for the field-off (field-on) data subsample. After requiring all relevant subdetectors to be performing normally, the subsamples used in the analysis of the field-off and field-on data contained approximately 1.6 million and 3 million minimum-bias events, respectively.

Monte Carlo (MC) event samples were used to determine the tracking efficiencies and the rates of fake tracks. The HIJING event generator [25] was used to produce minimum-bias Pb+Pb collisions. Events were generated with the default parameters except for jet quenching, which was turned off. The effect of elliptic flow was implemented after event generation. The azimuthal angles of final-state particles were redistributed at generator level to produce an elliptic flow consistent with previous ATLAS measurements [18, 19]. The simulation of the ATLAS detector’s response [26] to the generated events is based on the GEANT4 package [27] and included a detailed description of the detector geometry and material in the 2010 Pb+Pb run. Two samples of 0.5 million MC events were simulated, one with the solenoid field switched off and the other with it switched on. Additional MC samples consisting of 50,000 events simulated with 10–20 % extra detector material were used to study systematic uncertainties. The generated charged particles were reweighted with $p_{\mathrm {T}}$- and centrality-dependent functions so that the $p_{\mathrm {T}}$ spectra in the MC samples matched the experimental ones [28].

The centrality of the Pb+Pb collisions was characterized by the summed transverse energy, $\varSigma E_{\mathrm {T}}^{\mathrm {FCal}}$, measured in the FCal [18]. The $\varSigma E_{\mathrm {T}}^{\mathrm {FCal}}$ distribution was divided into ten centrality bins, each representing 10 % of the full distribution after accounting for 2 % inefficiency in recording the most peripheral collisions (the 0–10 % centrality interval corresponds to the most central 10 % of collisions: those with the largest $\varSigma E_{\mathrm {T}}^{\mathrm {FCal}}$). A small change in the overall recording efficiency leads to large fluctuations in the population of the most peripheral collisions. To avoid resulting large systematic uncertainties, the 20 % of events with the smallest $\varSigma E_{\mathrm {T}}^{\mathrm {FCal}}$ were not included in the analysis.

## Elliptic-flow measurement

The final-state azimuthal anisotropy is quantified by the coefficients in the Fourier expansion of the $\phi$ distribution of charged particles [3],

1 $$\begin{aligned} {\mathrm {d}}N/{\mathrm {d}}\phi \propto 1+2\sum _{n=1}^{\infty }v_n\cos {(n[\phi -\varPsi _n])}, \end{aligned}$$

where $v_n$ and $\varPsi _n$ are the magnitude and the azimuthal direction (called the event-plane angle) of the $n$-th flow harmonic, respectively.

The second flow harmonic, $v_2$, for a given collision centrality is a function of $p_{\mathrm {T}}$ and $\eta$, and is determined by

2 $$\begin{aligned} v_2(\eta ,p_{\mathrm {T}}) =\frac{\langle \cos {(2[\phi -\varPsi _2])}\rangle }{\sqrt{\langle \cos {(2[\varPsi _2^N-\varPsi _2^P])}\rangle }}, \end{aligned}$$

where the numerator denotes the average over charged-particle tracks in a given $\eta$ and $p_{\mathrm {T}}$ range, and the denominator, averaged over events, is a correction accounting for the finite experimental resolution in the determination of the event-plane angle $\varPsi _2$. This resolution correction was obtained using the sub-event technique [3] as described in Refs. [18, 19]. The two “sub-events” defined for each event cover two $\eta$ ranges of the same width in the positive and negative $\eta$ hemispheres ($3.2<|\eta |<4.8$) of the FCal detector. The sub-event-plane angles are determined by

3 $$\begin{aligned} \varPsi _2^{{\mathrm {N(P)}}} = \frac{1}{2}\tan ^{-1}\left( \frac{\sum \limits _i E^{\mathrm {tower}}_{{\mathrm {T}}i} w_i\sin (2\phi _i)}{\sum \limits _i E^{{\mathrm {tower}}}_{{\mathrm {T}}i} w_i\cos (2\phi _i)}\right) , \end{aligned}$$

where the sums run over transverse energies, $E_{\mathrm {T}}^{\mathrm {tower}}$, as measured in calorimeter towers located at negative (N) and positive (P) $\eta$ in the first sampling layer of the FCal. The FCal towers consist of cells grouped into projective regions in $\varDelta \eta \times \varDelta \phi$ of $0.1 \times 0.1$. The weights, $w_i(\varDelta \eta _i,\varDelta \phi _i)$ are used to correct for any non-uniformity in the event-averaged azimuthal angle distribution of $E_{\mathrm {T}}^{\mathrm {tower}}$. They are determined from the data in narrow $\varDelta \eta _i$ and $\varDelta \phi _i$ slices.

In the sub-event approach, potential non-flow correlations are minimized by using the reaction plane estimated from the $\eta$ side opposite to the tracks used for the $v_2$ measurement; this provides a separation of $\varDelta \eta > 3.2$. This method was previously applied [18] to measure $v_2$ as a function of $p_{\mathrm {T}}$ using charged-particle tracks reconstructed in the ID tracking system with a minimum $p_{\mathrm {T}}$ of 0.5 GeV.

In order to perform the integration over $p_{\mathrm {T}}$, the differential $v_2$ measurements are weighted by the number of charged-particle tracks $N_{i,k}^{\mathrm {corr}}$,

4 $$\begin{aligned} v_2= \sum _i \sum _kv_2(\eta _i,p_{\mathrm {T},k}) N_{i,k}^{{\mathrm {corr}}}/\sum _i \sum _k{N_{i,k}^{{\mathrm {corr}}}}, \end{aligned}$$

and summed over bins in $\eta$ (denoted by the index $i$) and $p_{\mathrm {T}}$ (index $k$). The number of charged-particle tracks is calculated as $N_{i,k}^{{\mathrm {corr}}} =N_{i,k}[1-f(i,k)]/\epsilon (i,k),$ where the $N_{i,k}$ is the observed number of tracks in a given $\eta$ and $p_{\mathrm {T}}$ bin, $\epsilon (i,k)$ is the track reconstruction efficiency and $f(i,k)$ is the estimated rate of fake tracks. In the following sections, the lower limit in the integration of $v_2$ over $p_{\mathrm {T}}$ is denoted by $p_{\mathrm {T,0}}$.

## Track reconstruction

The ID was used to reconstruct charged-particle trajectories. Three track reconstruction methods were applied in order to exploit a large range in particle $p_{\mathrm {T}}$:

- the tracklet (TKT) method used for the field-off data in order to reach charged-particle $p_{\mathrm {T}}$ below 0.1 GeV [28],
- the pixel track (PXT) method used to reconstruct tracks with $p_{\mathrm {T}}\ge 0.1$ GeV using only the pixel detector in the field-on data sample,
- the ID track (IDT) method for the field-on data sample, the default ATLAS reconstruction method, for which all ID sub-detectors are used and the track $p_{\mathrm {T}}$ is limited to $p_{\mathrm {T}}\ge 0.5$ GeV [29].

**Fig. 1:**
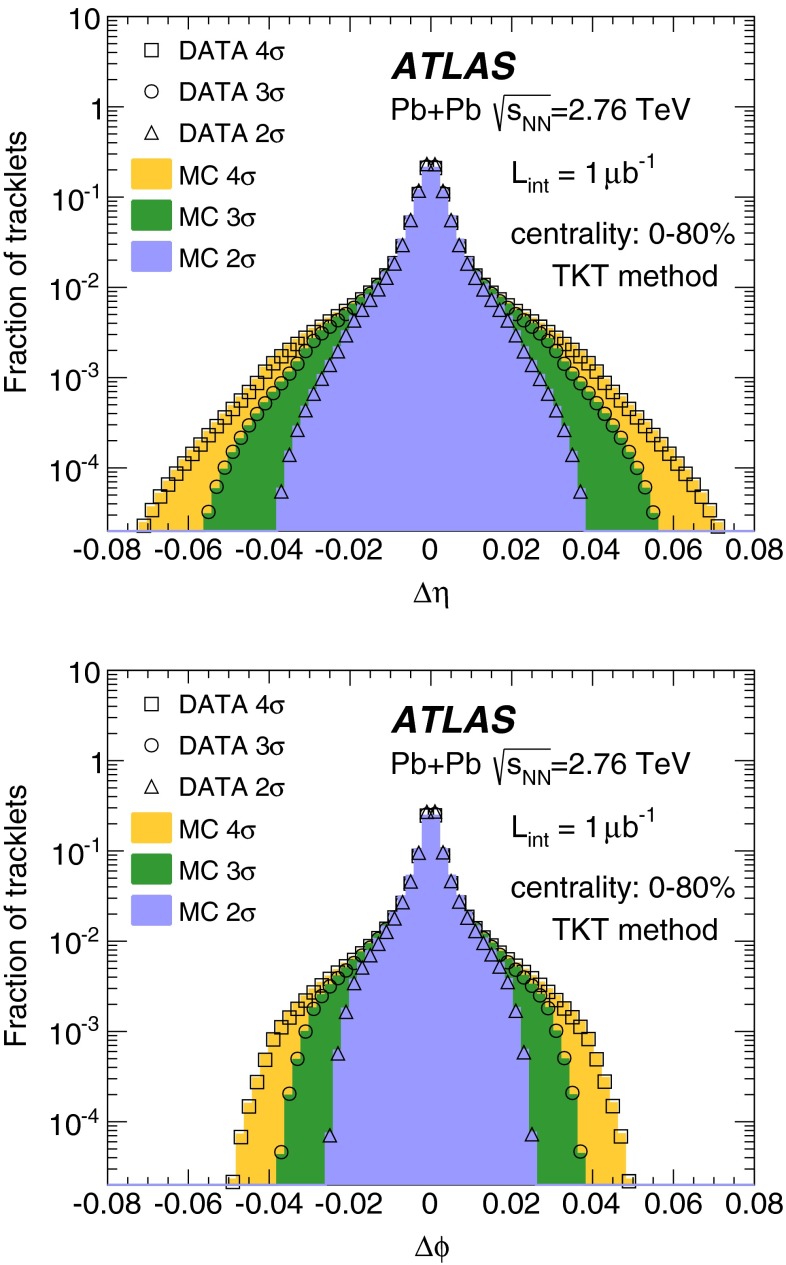
Comparison of the tracklets’ $\varDelta \eta$ (*top*) and $\varDelta \phi$ (*bottom*) distributions in data (*open symbols*) and MC simulation (*filled histograms*) for tracklets measured within the pseudorapidity range $|\eta |<2$, for events in the 0–80 % centrality interval and $\varDelta R < 4 \sigma , 3 \sigma$ and $2\sigma$ (see Sect. 5 for details) as described in the legend

**Fig. 2:**
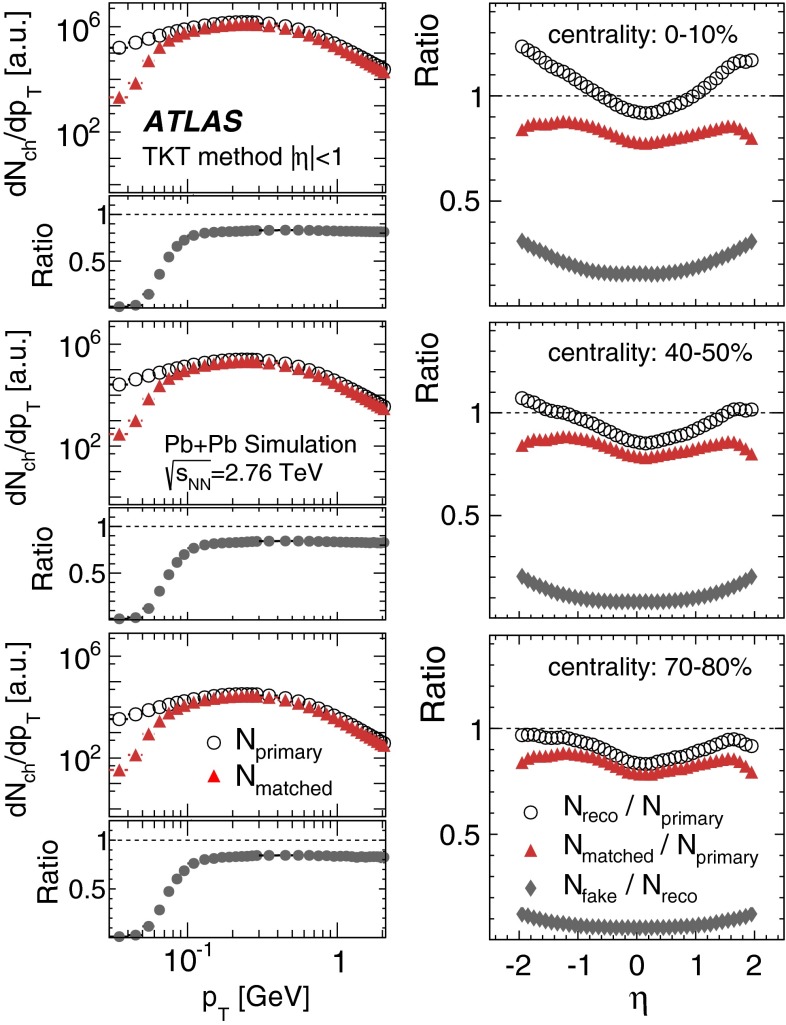
Monte Carlo evaluation of the tracklet reconstruction performance in representative centrality bins 0–10, 40–50 and 70–80 %. *Left* generator-level transverse momentum distributions of primary charged particles, $N_{{\mathrm {primary}}}$ (*open circles*), compared to the $p_{\mathrm {T}}$ spectra of charged particles matched to the reconstructed tracklets, $N_{{\mathrm {matched}}}$ (*red triangles*). *Bottom panels* show the ratios of the two distributions. *Right* pseudorapidity, $\eta$, dependence of the ratio of all reconstructed tracklets, $N_{{\mathrm {reco}}}$ (*open circles*), and $N_{{\mathrm {matched}}}$ (*red triangles*) to all primary charged particles. The ratio of fake tracklets, $N_{{\mathrm {fake}}}$ (*grey diamonds*), to all reconstructed tracklets is also shown

In the TKT method for field-off data, tracks are formed from the positions of hit clusters in the inner two layers of the pixel detector and the position of the primary vertex reconstructed using ID tracks. In the first step, the $\eta _0$ and $\phi _0$ coordinates are defined using the event’s vertex position and the hit recorded in the first pixel layer. Then a search for a hit in the second pixel layer (with $\eta _1$ and $\phi _1$ coordinates defined with respect to the vertex position) is performed and its consistency with a straight-track hypothesis is checked. Candidate tracklets are required to satisfy the condition

5 $$\begin{aligned} \varDelta R = \frac{1}{\sqrt{2}} \sqrt{\left( \frac{\varDelta \eta }{\sigma _{\eta }(\eta _0)} \right) ^{2} + \left( \frac{\varDelta \phi }{\sigma _{\phi }(\eta _0))} \right) ^{2}} < N_{\sigma }, \end{aligned}$$

where $\varDelta \eta =\eta _{1}-\eta _{0}$ and $\varDelta \phi =\phi _1-\phi _0$, and $\sigma _{\eta }(\eta _0)$ and $\sigma _{\phi }(\eta _0)$ are pseudorapidity-dependent widths of the $\varDelta \eta$ and $\varDelta \phi$ distributions, respectively. In this analysis, $N_{\sigma }=3$ was used as the default condition. Clusters located close to each other in the second pixel layer are most likely to originate from the same particle. Therefore, if more than one cluster located in the second pixel layer fulfils the selection criteria, the resulting tracklets are merged into a single tracklet. The $\varDelta \eta$ and $\varDelta \phi$ distributions in data and MC simulation are compared in Fig. 1. The data and MC distributions agree well. Candidates fulfilling the criterion in Eq. (5) were accepted for further analysis with $\eta =\eta _0$ and $\phi =\phi _0$.

**Fig. 3:**
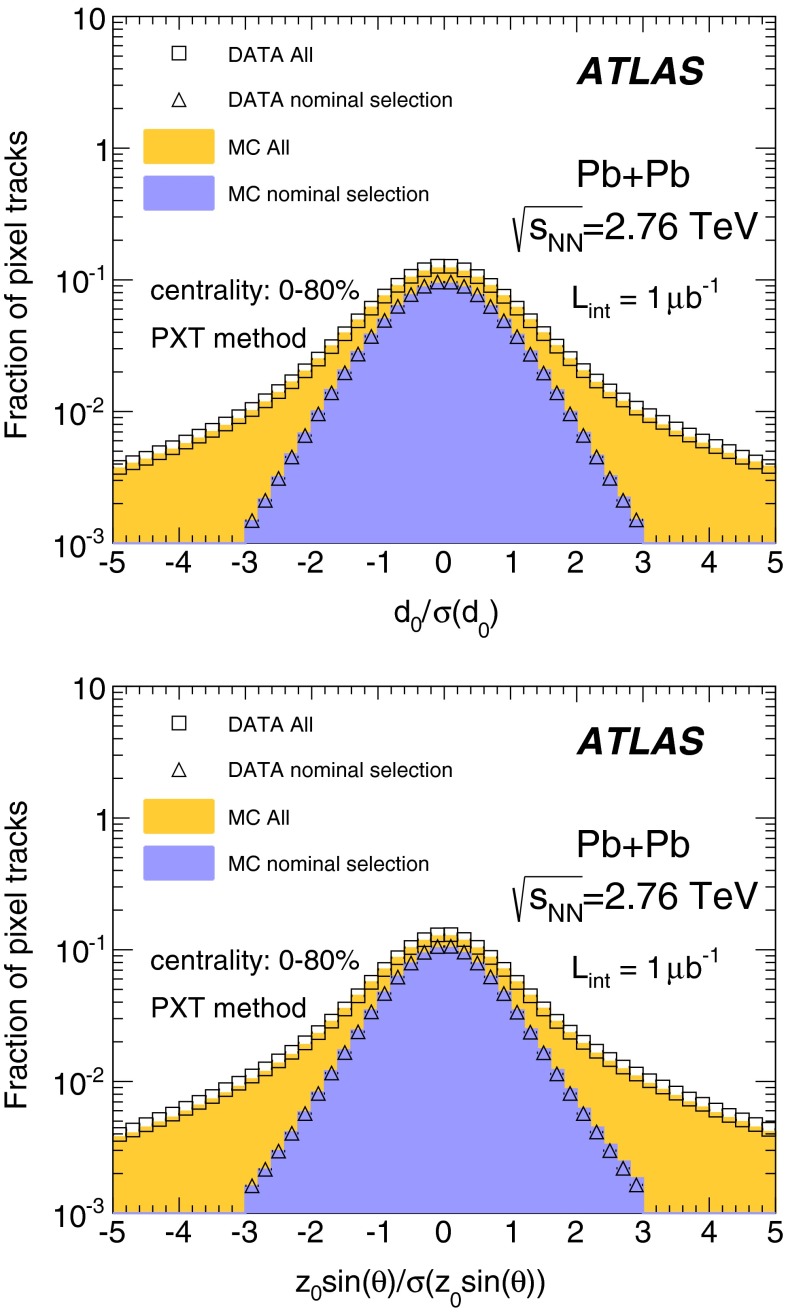
Comparison of distributions of the transverse (*top*), and longitudinal (*bottom*) impact parameter significances in data and MC simulation for all reconstructed tracks and for the selected tracks (see text for details)

This method does not provide information about the track’s $p_{\mathrm {T}}$; nevertheless, its performance can be checked as a function of generator-level particle $p_{\mathrm {T}}$ by applying the same reconstruction procedure to the MC simulation and using the $p_{\mathrm {T}}$ of the generated particle corresponding to the reconstructed tracklet whenever applicable. Figure 2 compares the $p_{\mathrm {T}}$ spectra of stable charged particles at the MC-generator level, $N_{{\mathrm {primary}}}$, to the spectra of reconstructed tracklets matched to charged particles, $N_{{\mathrm {matched}}}$, for three representative centrality bins and for $|\eta | < 1$. A particle was considered to be primary if it originated directly from the collision or resulted from the decay of a particle with $c\tau <1$ mm. The matching criterion required that the two hits forming the tracklet be identical to the hits associated with a charged particle. The distributions show that the TKT method is able to reconstruct particles with transverse momenta ${\sim }0.07$ GeV with 50 % efficiency, and that a plateau at about 80 % is reached for $p_{\mathrm {T}}> 0.1$ GeV in all centrality bins. For low $p_{\mathrm {T}}$, the efficiency decreases sharply, but the particle density is small in this region, as is $v_2$; thus the contribution from this region to the integrated elliptic flow is expected to be small. Figure 2 also shows the reconstruction efficiency, $N_{{\mathrm {matched}}}/N_{{\mathrm {primary}}}$, as a function of $\eta$. Here, $N_{{\mathrm {primary}}}$ denotes all primary charged particles with $p_{\mathrm {T}}\ge 0.07$ GeV, which defines the low-$p_{\mathrm {T}}$ limit for integrating $v_2$ over $p_{\mathrm {T}}$. The efficiency is found to be ${\sim }80$ % and depends weakly on $\eta$. The rate of fake tracklets, $N_{{\mathrm {fake}}}$, measured as the ratio of the number of tracklets not matched to charged particles to the total number of reconstructed tracklets, $N_{{\mathrm {fake}}}/N_{{\mathrm {reco}}}$, increases with centrality and $|\eta |$, reaching about 35 % for the most central collisions at $|\eta |=2$. For field-on data, the PXT method allows the transverse momentum range $p_{\mathrm {T}}> 0.1$ GeV to be examined. Tracks were reconstructed within the full acceptance of the pixel detector ($|\eta |<2.5$). To improve the track reconstruction’s performance in the heavy-ion collision environment, the track-quality requirements were made more stringent than those for proton–proton collisions [30]. Pixel tracks were required to have no missing hits in the pixel layers, and the transverse and longitudinal impact parameters, $d_0$ and $z_0$, with respect to the vertex were required to have $|d_0|$ and $|z_0\sin (\theta )|$ less than 1 mm and significances $|d_0/\sigma _{d_0}|$ and $|z_0\sin {\theta }/\sigma _{z_0\sin {\theta }}|$ less than 3.0. Figure 3 shows good agreement between data and MC simulation in the distributions of $|d_0/\sigma _{d_0}|$ and $|z_0\sin {\theta }/\sigma _{z_0 \sin {\theta }}|$.

**Fig. 4:**
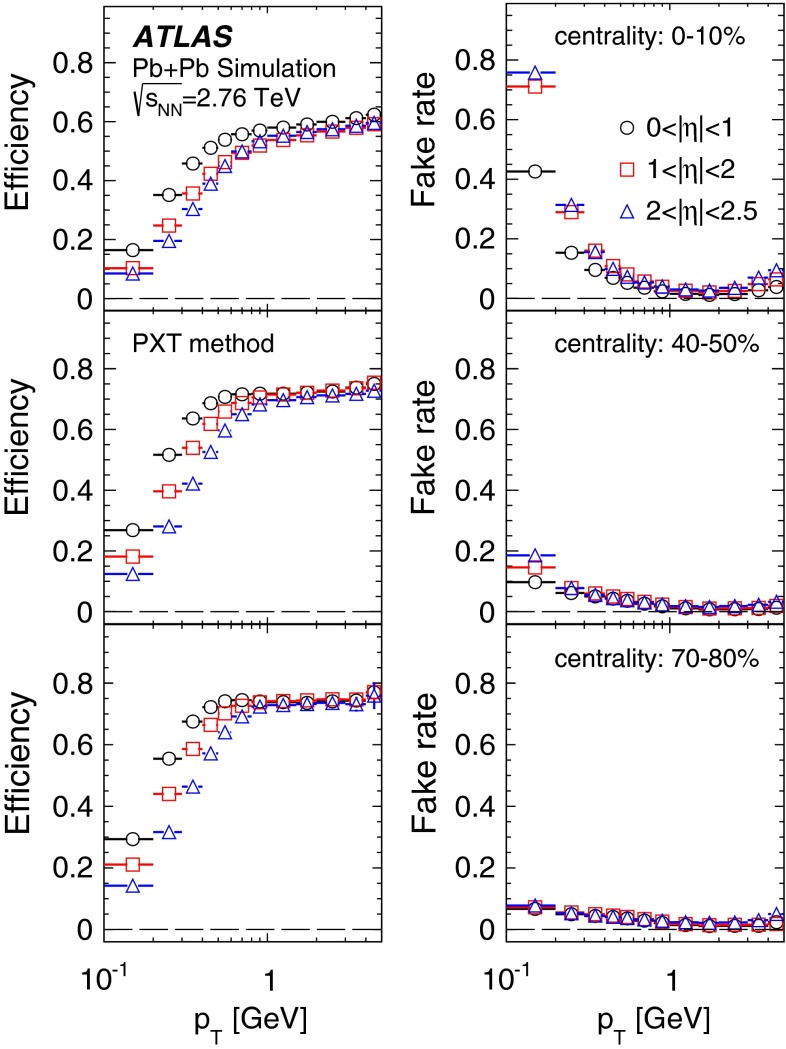
The transverse momentum, $p_{\mathrm {T}}$, dependence of the pixel track reconstruction efficiency (*left*) and the fake rate (*right*) for three pseudorapidity ranges and three centrality intervals as indicated in the legend

The pixel track method’s reconstruction efficiency was evaluated in MC simulation by matching reconstructed tracks to the generated charged particles. A track is matched to a generated charged particle if it is reconstructed from at least 69 % of the pixel hits originating from the latter. Figure 4 illustrates the dependence of the pixel track reconstruction efficiency on $p_{\mathrm {T}}$ in three pseudorapidity ranges and for three selected centrality bins. The efficiency decreases slightly from peripheral to central collisions and also deteriorates when moving away from mid-rapidity. A weak $p_{\mathrm {T}}$ dependence is observed above $p_{\mathrm {T}}> 0.5$ GeV for all collision centralities. At lower $p_{\mathrm {T}}$, the efficiency decreases with decreasing $p_{\mathrm {T}}$ and to about 20 % at the lowest accessible $p_{\mathrm {T}}$.

The fraction of fake tracks, defined as the ratio of reconstructed tracks not matched to generated charged particles to all reconstructed pixel tracks, was evaluated using MC simulation. Figure 4 shows the fake-rate dependence on $p_{\mathrm {T}}$ in three pseudorapidity ranges and for three centrality bins. The fake rate is below 10 % for $p_{\mathrm {T}}$ above 0.4 GeV and depends very weakly on $p_{\mathrm {T}}$ and $\eta$ for peripheral collisions. In more central collisions, the fake rate increases at low $p_{\mathrm {T}}$ and shows a similar increase with increasing $|\eta |$.

**Fig. 5:**
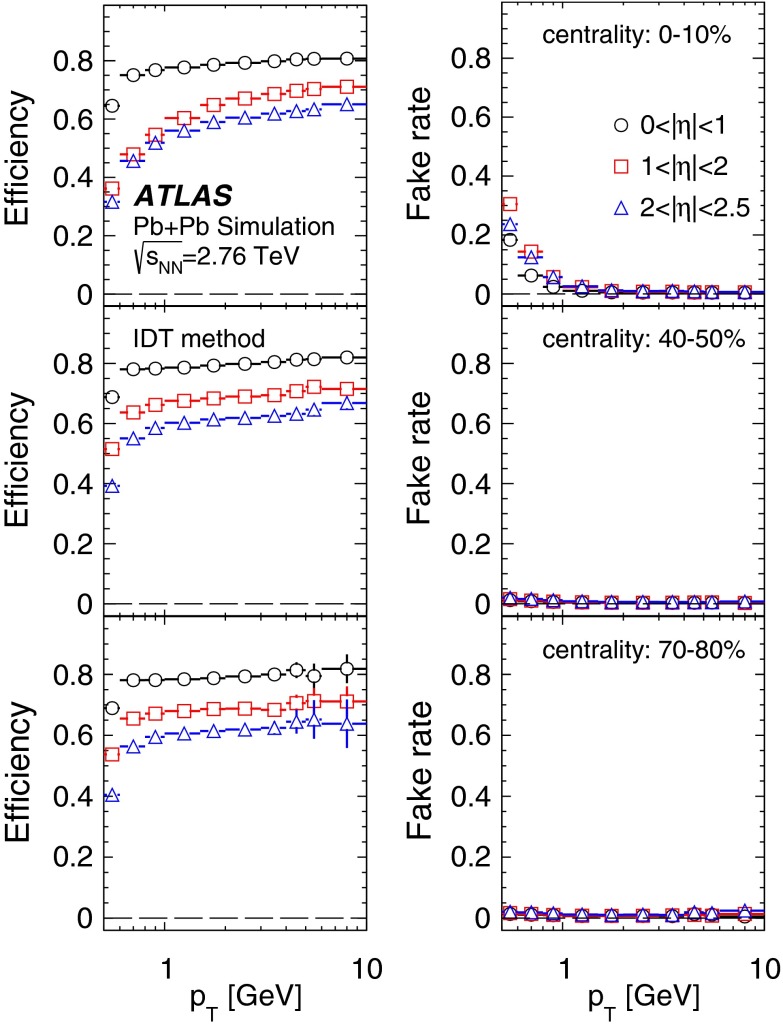
The transverse momentum, $p_{\mathrm {T}}$, dependence of the ID track reconstruction efficiency (*left*) and the fake rate (*right*) for three pseudorapidity ranges and three centrality intervals as indicated in the legend

The performance of the PXT reconstruction method can be compared with that of the IDT method. The track reconstruction efficiency and rate of fake tracks from the IDT method are shown in Fig. 5 (for reconstruction details see Ref. [18]). The minimum $p_{\mathrm {T}}$ reached is 0.5 GeV. A comparison of Figs. 4 and 5 shows that the extension towards lower $p_{\mathrm {T}}$ values for the PTX method is achieved at the expense of much larger fake rates than observed for the IDT method, whereas the reconstruction efficiencies are similar. The two methods have different $p_{\mathrm {T}}$ resolutions: it is very good for ID tracks, the root mean square of $(p_{\mathrm {T}}^{{\mathrm {reco}}}/p_{\mathrm {T}}^{{\mathrm {true}}}-1)$ being, in $|\eta |<1$, about 4 % and independent of the track $p_{\mathrm {T}}$ in the used range, whereas for pixel tracks it is about 10 % at the lowest $p_{\mathrm {T}}$ and increases to about 15 % at 5 GeV.

**Fig. 6:**
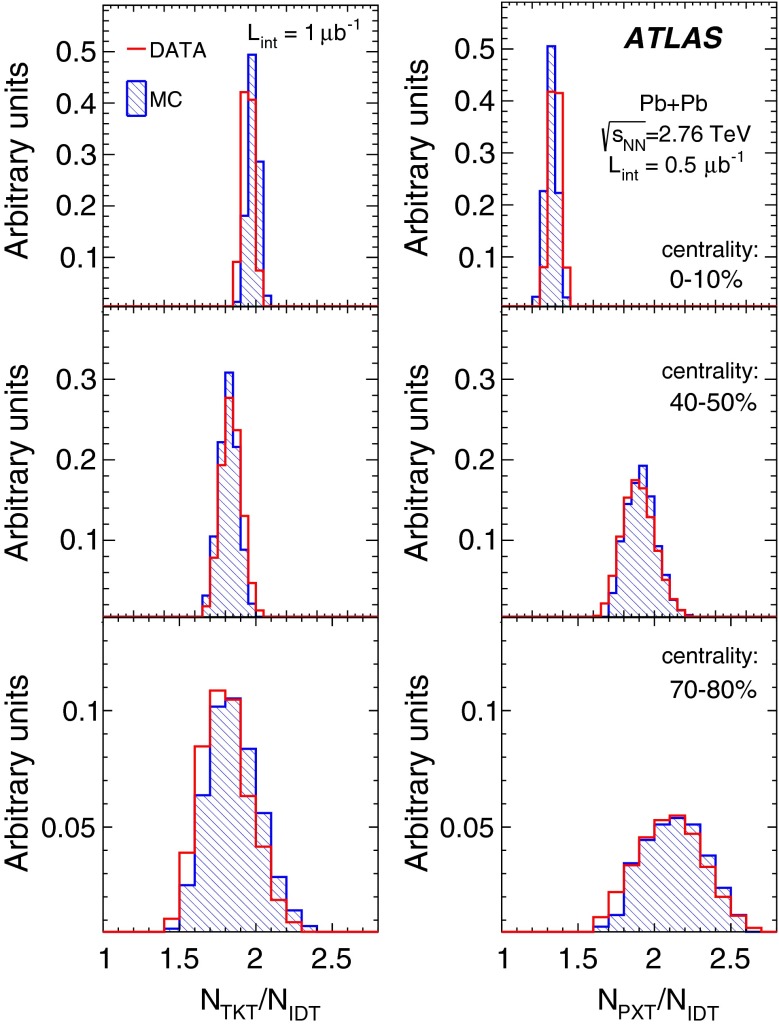
Comparison of the distribution of multiplicity ratios of number of tracklets, $N_{\mathrm {TKT}}$, (*left*) and pixel tracks, $N_{\mathrm {PXT}}$, (*right*) to the number of ID tracks, $N_{\mathrm {IDT}}$, in data (*red*) and MC simulation (*blue*) in three centrality bins as indicated on the plots

The performance of the MC simulation in describing the fake rates in the data was checked by comparing the $\varDelta \eta$, $\varDelta \phi$, $d_0/\sigma _{d_0}$ and $z_0 \sin {\theta }/\sigma _{z_0\sin {\theta }}$ distributions, like the ones shown in Figs. 1 and 3. Additionally, the distributions of the ratios of the number of tracklets and pixel tracks to the number of ID tracks in data and MC simulation were compared, as shown in Fig. 6. It can be concluded that the MC description of the TKT and PXT methods’ performance is adequate.

The elliptic flow depends on the particle type [31] as does the reconstruction efficiency. Although the track reconstruction efficiency is averaged over all particle types in this analysis, the reconstruction efficiencies for simulated pions, kaons and protons are shown as a function of $p_{\mathrm {T}}$ in the Appendix. At low transverse momenta, which are the focus of this analysis, the measured $v_2$ is dominated by pions with negligible contributions from kaons and protons. Nevertheless, the information on the particle type-dependent efficiencies can be used for detailed comparison of the measurement to theoretical predictions of the elliptic flow for identified particles.

## Corrections to measured $v_2$

The event-plane method [3] is applied to measure the differential elliptic flow harmonic $v_2(\eta )$ in small $\eta$ bins with the TKT method, and $v_2(\eta ,p_{\mathrm {T}})$ in small $\eta$ and $p_{\mathrm {T}}$ bins with the PXT and IDT methods. The differential $v_2$ measurements are then corrected for detector-related effects.

The first correction is associated with the variation in tracking efficiency induced by the flow itself. It is applied only to the PXT method, which is found to be sensitive to the detector occupancy. Such sensitivity is not observed for the IDT method. Since the flow phenomenon is a modulation of the multiplicity, it may induce a variation of the tracking efficiency in an event. Higher occupancy causes lower efficiency, and the number of tracks observed in the event plane is reduced more strongly than the number of tracks observed in other directions. As a consequence, the observed $v_2$ is smaller. In order to correct for this effect, an appropriate weight was applied to the tracks in the calculation of the numerator of Eq. (2). This weight, the inverted efficiency parameterized as a function of detector occupancy in the vicinity of the track, was derived from MC simulation. In the data, the occupancy was determined for each track from the number of hits near the track in the first layer of the pixel detector. The corrected $v_2(p_{\mathrm {T}})$ was compared to the measurement obtained from the IDT method in the same data. In the MC simulation, the comparison was made to $v_2(p_{\mathrm {T}})$ determined using generated particles. The relative increases in the value of $v_2(p_{\mathrm {T}})$ in data and in simulation were found to be compatible for $p_{\mathrm {T}}> 0.5$ GeV, the range in which the comparison could be performed.

The occupancy correction results in an increase of about 12 % in the integrated $v_2$ for the 0–20 % centrality interval while it amounts to only 1 % for the most peripheral collisions, when using a lower $p_{\mathrm {T}}$ integration limit of $p_{\mathrm {T,0}}= 0.1$ GeV. For higher values of $p_{\mathrm {T,0}}$ the correction gradually becomes smaller. For $p_{\mathrm {T,0}}= 0.5$ GeV it decreases to about 7 % for the most central collisions.

An additional correction, applied to the differential measurement of $v_2$, accounts for the difference between $v_2$ measured only with fake tracks and $v_2$ measured with charged-particle tracks from the primary vertex. The corrected $v_2$ is calculated as

6 $$\begin{aligned} v_2= \frac{v_2{}_{,m} - fv_2{}_{,f}}{1-f}, \end{aligned}$$

where $v_2{}_{,m}$ is the elliptic flow measured with all tracks, $v_2{}_{,f}$ is the flow of fake tracks, and $f$ is the fake-track rate. This correction was applied to the differential $v_2$ measured with the TKT, PXT and IDT methods with the corresponding fake rates and $v_2{}_{,f}$ values. The rate and $v_2{}_{,f}$ of the fake tracks were derived from MC simulation and then cross-checked in the data with a sample, obtained with inverted track selection criteria, in which fake tracks dominate. Differences between the MC simulation and the data of up to 20 % were observed and included in the systematic uncertainties.

The fake tracks reduce the values of $v_2$ integrated over the $p_{\mathrm {T}}$ ranges considered in this analysis. The correction is a function of the fake-track rate and accordingly exhibits a dependence on centrality, $p_{\mathrm {T}}$ and $\eta$. For $|\eta |<1$, the largest correction, about 15 %, was obtained for the PXT method with $p_{\mathrm {T,0}}= 0.1$ GeV. For peripheral collisions in the same kinematic range, it decreases to about 11 %. The correction is smaller for higher values of $p_{\mathrm {T,0}}$. It decreases to about 2 % for $p_{\mathrm {T,0}}= 0.5$ GeV for the 0–10 % centrality interval and gradually drops to zero for the most peripheral collisions. The fake-track flow correction for the integrated $v_2$ obtained with the IDT method ($p_{\mathrm {T,0}}= 0.5$ GeV) is less than 2 % for the most central collisions and even smaller for the more peripheral ones. For the TKT method, the correction is about 1 % for the most central collisions.

The limited $p_{\mathrm {T}}$ resolution for tracks reconstructed in the pixel detector and the rapidly changing ${\mathrm {d}}N_{\mathrm {ch}}/{\mathrm {d}}p_{\mathrm {T}}$ distribution lead to a significant bin-to-bin migration in $p_{\mathrm {T}}$. As a consequence of the variation of $v_2$ with $p_{\mathrm {T}}$, $v_2$ measured in a given $p_{\mathrm {T}}$ bin is contaminated by $v_2$ values of particles from the neighbouring bins. In order to compensate for this effect, a correction derived from MC simulation was applied to the $v_2(p_{\mathrm {T}})$ values. This correction was determined, using pixel tracks matched to generated particles, by comparing the $v_2(p_{\mathrm {T}})$ distribution as a function of reconstructed $p_{\mathrm {T}}$ to $v_2(p_{\mathrm {T}})$ as a function of generated $p_{\mathrm {T}}$. In order to validate the correction derived from the MC simulation, the same procedure was applied in the data and in the simulation in the region of $p_{\mathrm {T}}> 0.5~\hbox {GeV}~$, where the ID tracks were used instead of the generated particles. The ID tracks were matched by requiring an angular separation $\sqrt{(\varDelta \eta )^2 + (\varDelta \phi )^2} < 0.02$. A comparison between the corrections obtained in the data and in the MC simulation, as a function of measured $p_{\mathrm {T}}$, showed a good agreement.

The correction for $p_{\mathrm {T}}$-bin migration of the reconstructed tracks was found to be small compared to the occupancy and fake-track flow corrections, and to depend only on the value of $p_{\mathrm {T,0}}$. It increases the integrated $v_2$ value by 1 % (1.5 %) for $p_{\mathrm {T,0}}= 0.1$ GeV ($p_{\mathrm {T,0}}= 0.5$ GeV) independently of collision centrality.

**Fig. 7:**
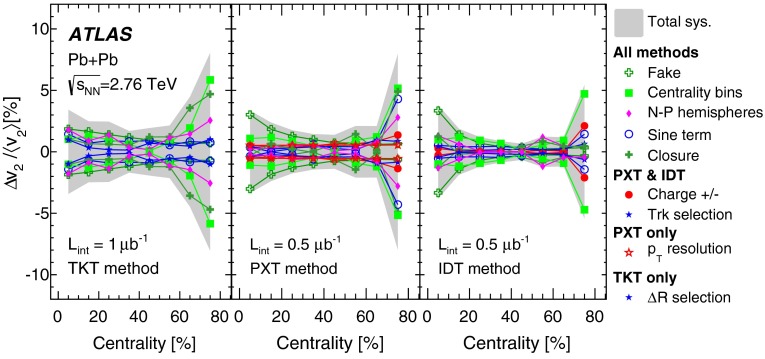
Contributions to the relative systematic uncertainty on the elliptic flow, $\varDelta v_2/ \langle v_2\rangle$, as a function of centrality for $|\eta |<1$ with the TKT (*left*), PXT (*centre*) and IDT (*right*) methods. The integration limits for the three methods are 0.07, 0.1, 0.5 GeV, respectively. The total uncertainty is indicated by the *shaded area*. The individual contributions, are described in the legend and explained in the text

## Uncertainties in the $v_2$ determination

The systematic uncertainties include those common to different tracking methods, as well as method-specific ones.

The uncertainty which originates from the statistics of the MC samples is treated as a source of systematic uncertainty.

The $v_2$ values determined for samples enriched in fake tracks in data and MC simulation were compared and differences of up to 20 % for both the PXT and IDT methods were observed. For the PXT method, this difference resulted in a change of $v_2$, integrated from $p_{\mathrm {T,0}}= 0.1$ GeV, for the most central (0–10 %) collisions of 3 % at mid-rapidity and of 15 % at $|\eta |\sim 2$. The impact on the integrated $v_2$ decreases with increasing centrality. For higher $p_{\mathrm {T,0}}$ values, the change was found to be negligible. For the IDT method, the uncertainty on the $v_2$ value of fake tracks induces a systematic uncertainty in the integrated $v_2$ for central collisions of less than 4 % at mid-rapidity and of 9 % at $|\eta |\sim 2$; for peripheral collisions the uncertainty is smaller.

The variation of the fake tracklets’ $v_2$, at the level of 10 %, obtained from the comparison of data and MC simulation, results in an uncertainty at the level of 2 % in the integrated $v_2$ across the centrality range 0–40 %.

A comparison of $v_2$ values obtained with the TKT method for a MC sample with the nominal detector geometry to that with 10 % more active material and 15–20 % more inactive material shows agreement to better than 2 %. Therefore it was assumed that possible inaccuracies in the description of the detector material in the GEANT4 simulation have a negligible effect on the final results. The same holds for the measurements with the PXT and IDT methods.

An overall scale uncertainty on $v_2$ originates from the uncertainty on the fraction of the total inelastic cross section accepted by the trigger as well as from the event selection criteria, which affects the population of the centrality bins. It is negligibly small (below 1 %) for central collisions and increases to about 6 % for the most peripheral collisions for the TKT method and to about 5 % for both the PXT and IDT methods.

The influence of the detector nonuniformities on the measured $v_2$ was checked by comparing the $v_2$ values obtained for positive and negative $\eta$. This led to a typical uncertainty of 1 % except for the most peripheral collisions where it increased to about 2 %.

**Table 1:** Summary of the systematic uncertainties as percentages of the integrated $v_2$ value for charged particles with $|\eta |<1$ and different collision centrality bins

Source	Centrality bin				
	0–10 %	10–20 %	20–60 %	60–70 %	70–80 %
	TKT $p_{\mathrm {T}}> 0.07$ GeV				
MC Statistics	0.1	0.1	$<$0.2	0.3	1
Fake tracks	2	2	1–2	1	1
Centrality bins	1	1.5	$<$1	2	6
N-P $\eta$ regions	2	1	$<$1.5	1	2.5
Sine term	1.5	1	1	1	1
Closure	1.5	1	$<$2	3.5	5
$\varDelta R$	1	0.5	$<$1	0.5	1
Total	3.5	3.2	$<$3.2	4	8
	PXT $p_{\mathrm {T}}> 0.1$ GeV				
MC Statistics	0.1	0.1	$<$0.2	0.3	1
Fake tracks	3	2	$<$1.5	0.5	0.5
Centrality bins	1	1.5	$<$1	1.5	5
N-P $\eta$ regions	0.5	0.5	$<$0.5	1	3
Sine term	0.5	0	$<$0.5	1	4
Closure	1	1	$<$2	0	5
Charge $\pm$	0.5	0.5	$<$1	1	1.5
Track selection	0.5	0.5	$<$0.5	1	1
$p_{\mathrm {T}}$ resolution	0.5	0.5	0.5	0.5	0.5
Total	3	2	$<$2	2	8
	IDT $p_{\mathrm {T}}> 0.5$ GeV				
MC Statistics	0.1	0.1	$<$0.2	0.3	1
Fake tracks	3.5	1.5	$<$1	0.2	0.2
Centrality bins	1	1.5	$<$1	1	5
N-P $\eta$ regions	1.2	1	$<$1.5	0.5	0.5
Sine term	0.5	0.5	0.5	0.5	1.5
Closure	1.5	0.5	$<$1	0.5	0.5
Charge $\pm$	0.2	0.2	0.2	0.2	2.2
Track selection	0.5	0	$<$0.5	0.2	1
Total	3.5	2	$<$1.5	1	5.5

Deviations of $\left<\sin {2[\phi -\varPsi _2]}\right>$ from zero point to detector non-uniformities and biases in the event-plane determination. The magnitude of the sine term relative to the cosine term is included in the systematic uncertainty of $v_2$. For the TKT method, its contribution to the relative systematic uncertainty is negligibly small. For the PXT and IDT methods, it is small for most centrality bins, and increases to 2 % only for the most peripheral collisions.

**Fig. 8:**
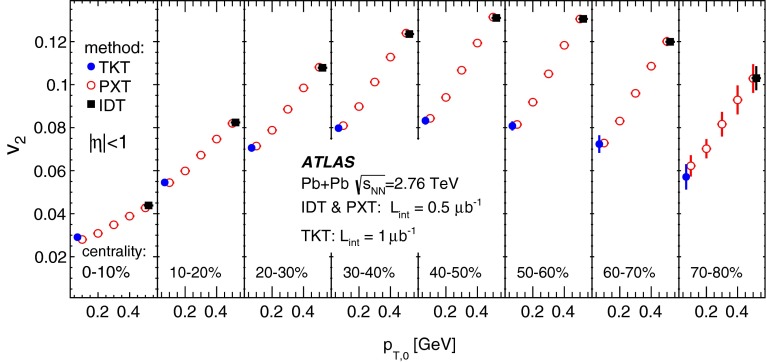
Elliptic flow $v_2$ integrated over transverse momentum $p_{\mathrm {T}}>p_{\mathrm {T,0}}$ as a function of $p_{\mathrm {T,0}}$ for different centrality intervals, obtained with different charged-particle reconstruction methods: the tracklet (TKT) method with $p_{\mathrm {T,0}}= 0.07$ GeV, the pixel track (PXT) method with $p_{\mathrm {T,0}}\ge 0.1$ GeV and the ID track (IDT) method with $p_{\mathrm {T,0}}= 0.5$ GeV as described in the legend. *Error bars* show statistical and systematic uncertainties added in quadrature

The analysis procedure was checked with MC studies in which the generated elliptic flow signal was compared to the $v_2$ values obtained with the same analysis chain as used for the data. In this MC closure test, relative differences of up to 2 % in central collisions and of up to 5 % in peripheral collisions were observed for the TKT method. For the IDT method, the relative difference reaches 2 %; for the PXT method, it remains within 2 % except for the most peripheral collisions where it increases to 5 %. The relative difference between the expected and measured values is included in the total systematic uncertainty.

The $\varDelta R$ parameter used in the tracklet reconstruction was varied by ${\pm }1 \sigma$ from the nominal value. The resulting variation in the value of $v_2$ at the level of 1 % is included in the systematic uncertainty. For the PXT and IDT methods, differences between $v_2$ determined from tracks of negatively and positively charged particles as well as between the baseline $v_2$ and that obtained with tighter or looser tracking requirements (in which the transverse and longitudinal impact parameter significance criteria are changed by $\pm 1$) also contribute to the systematic uncertainty at the level of a few percent.

For the PXT method, the corrections due to the limited $p_{\mathrm {T}}$ resolution were varied within their statistical uncertainties and the resulting variation was found to be at the level of 0.5 %, independently of the centrality.

The $p_{\mathrm {T}}$ spectrum of charged particles in the MC simulation was reweighted so that the expected detector-level distribution agrees with that observed in the data. This changes the effective fake-track rate and therefore the weights used in the calculation of $v_2$. A variation of these weights by up to 10 % has a negligible effect on the determination of $v_2$.

The different contributions to the total systematic uncertainty on the integrated $v_2$ for $|\eta |<1$ are shown in Fig. 7 and summarized in Table 1 for the three tracking methods. The total systematic uncertainties are determined by adding in quadrature all the individual contributions and are treated as ${\pm }1\sigma$ uncertainties.

**Fig. 9:**
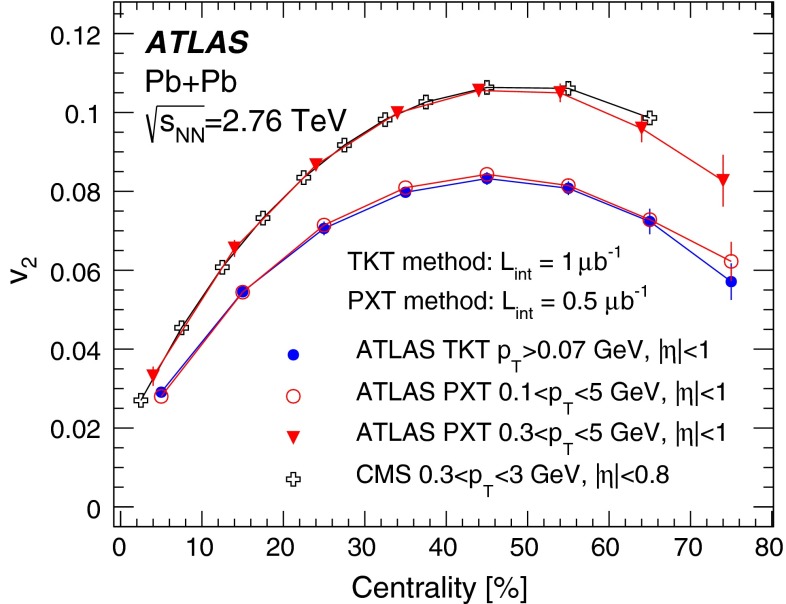
Centrality dependence of elliptic flow, $v_2$, measured for $|\eta |<1$ and integrated over transverse momenta, $p_{\mathrm {T}}$, for different charged-particle reconstruction methods as described in the legend. Also shown are $v_2$ measurements by CMS integrated over $0.3<p_{\mathrm {T}}<5$ GeV and $|\eta |<0.8$ [20] (*open crosses*). *Error bars* show statistical and systematic uncertainties added in quadrature

## Results

Figure 8 shows the centrality dependence of $v_2$ integrated over $|\eta |<1$. For the TKT method, $v_2$ is integrated over $p_{\mathrm {T}}>0.07$ GeV. For the PXT method, $v_2$ is integrated over $p_{\mathrm {T,0}}< p_{\mathrm {T}}< 5$ GeV and $p_{\mathrm {T,0}}$ is varied from 0.1 to 0.5 GeV in steps of 0.1 GeV. Also shown is the $v_2$ value obtained from the IDT method integrated over $0.5 < p_{\mathrm {T}}< 5$ GeV. The TKT method with $p_{\mathrm {T,0}}= 0.07$ GeV gives results consistent with the $v_2$ values obtained with the PXT method with $p_{\mathrm {T,0}}=0.1$ GeV, as could be expected due to the very low charged-particle density and small $v_2$ signal in the momentum range below 0.1 GeV. This indicates that there is no need to extrapolate the measurements obtained with tracklets down to $p_{\mathrm {T}}= 0$ in order to obtain a reliable estimate of $v_2$ integrated over the whole kinematic range in $p_{\mathrm {T}}$. Furthermore, for the PXT method such an extrapolation would result in a very small correction to the measured integrated flow, well within the uncertainties of the measurement. This is in contrast to the integrated $v_2$ with $p_{\mathrm {T,0}}$ chosen at higher values, as also shown in Fig. 8. It can be seen that the integrated $v_2$ increases almost linearly with $p_{\mathrm {T,0}}$ for $p_{\mathrm {T,0}}> 0.1$ GeV. Good agreement between the PXT and IDT methods is observed at $p_{\mathrm {T,0}}= 0.5$ GeV. In Fig. 9, the results of this analysis are compared to the integrated $v_2$ measured by CMS [20] with $p_{\mathrm {T,0}}= 0.3$ GeV. In this comparison, the sensitivity to $p_{\mathrm {T,0}}$ is clearly visible. A systematically larger $v_2$ is observed for the higher value of $p_{\mathrm {T,0}}$ as a consequence of the strong increase of $v_2$ with increasing $p_{\mathrm {T}}$.

**Fig. 10:**
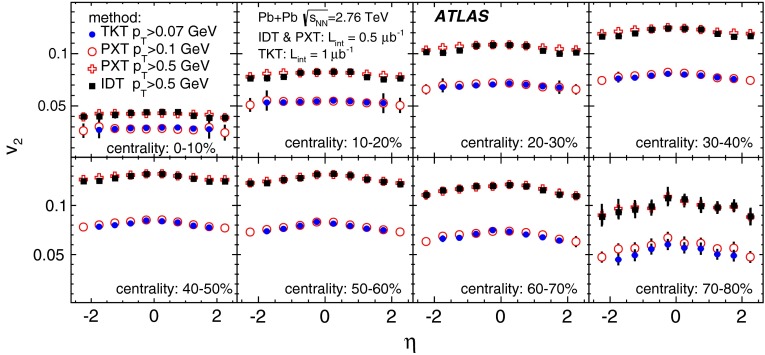
Pseudorapidity, $\eta$, dependence of elliptic flow, $v_2$, integrated over transverse momentum, $p_{\mathrm {T}}$, for different charged particle reconstruction methods and different low-$p_{\mathrm {T}}$ thresholds in different centrality intervals as indicated in the legend. *Error bars* show statistical and systematic uncertainties added in quadrature

**Fig. 11:**
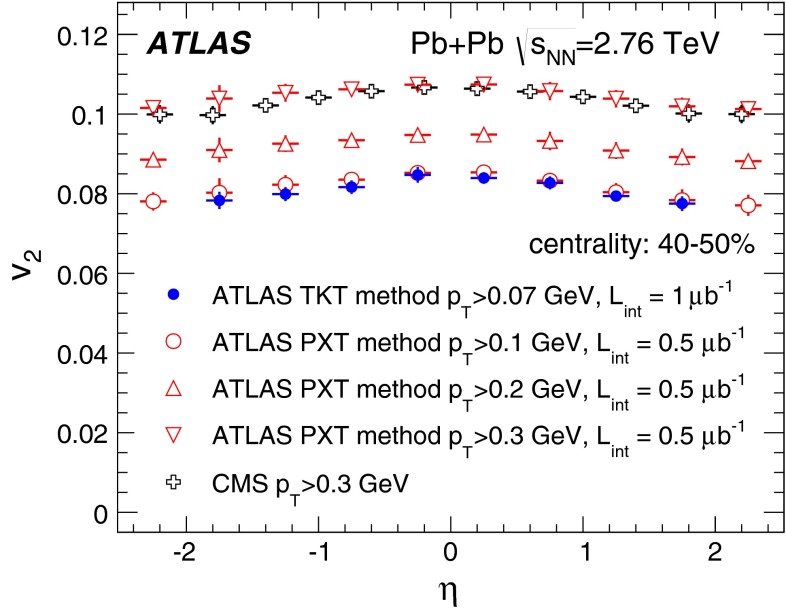
Comparison of the pseudorapidity, $\eta$, dependence of elliptic flow, $v_2$, integrated over transverse momentum, $p_{\mathrm {T}}$, for different low-$p_{\mathrm {T}}$ thresholds, as indicated in the legend, in the 40–50 % centrality interval from the ATLAS and CMS experiments. *Error bars* show statistical and systematic uncertainties added in quadrature

The $\eta$ dependence of the $p_{\mathrm {T}}$-integrated $v_2$ provides useful constraints on the initial conditions of heavy-ion collisions used in model descriptions of the system’s evolution (see, e.g., Refs. [1, 2]). Figure 10 shows the $\eta$ dependence of the $p_{\mathrm {T}}$-integrated $v_2$. As already shown in Fig. 9, the difference between the results obtained with $p_{\mathrm {T,0}}$ values of 0.07 and 0.1 GeV is very small and the two measurements agree within uncertainties. The results obtained using the PXT and IDT methods for the same $p_{\mathrm {T,0}}$ are also consistent. The $\eta$ dependence of the integrated $v_2$ is very weak. A decrease with increasing $|\eta |$ of about 5–10 % is seen. A comparison with the results from the CMS experiment [20] is shown in Fig. 11 for the 40–50 % centrality interval. The ATLAS measurements performed with the PXT method were integrated over $p_{\mathrm {T}}$ for different $p_{\mathrm {T,0}}$ values, including one adjusted to match that used by CMS. The results agree, within uncertainties, provided the same $p_{\mathrm {T,0}}$ is used.

The different upper limits in the $p_{\mathrm {T}}$ integration, 3 GeV for CMS and 5 GeV for ATLAS, have negligible effect on the integrated $v_2$ value. A systematic decrease in $v_2$ with decreasing $p_{\mathrm {T,0}}$ is observed as expected from the linear dependence of $v_2$ on $p_{\mathrm {T}}$ for $p_{\mathrm {T}}\approx 0$. The decreasing value of $p_{\mathrm {T,0}}$ together with that of $v_2$ makes the integration over the full $p_{\mathrm {T}}$ range less sensitive to the uncertainties in the extrapolation down to $p_{\mathrm {T}}= 0$.

**Fig. 12:**
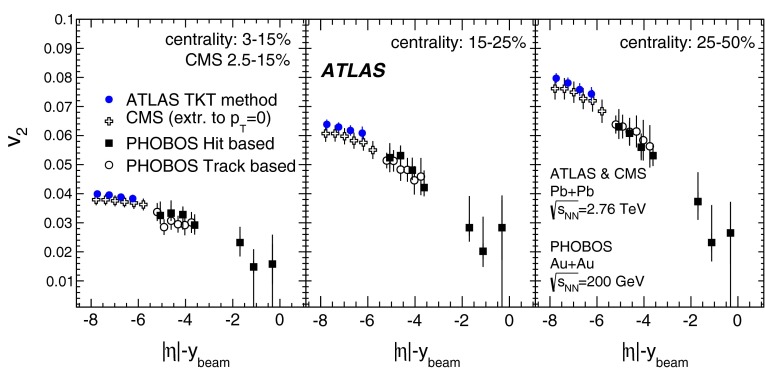
Integrated elliptic flow, $v_2$, as a function of $|\eta |-y_{{\mathrm {beam}}}$ for three centrality intervals indicated in the legend, measured by the ATLAS and CMS experiments for Pb+Pb collisions at 2.76 TeV and by the PHOBOS experiment for Au+Au collisions at 200 GeV. The CMS result is obtained by averaging the $v_2(p_{\mathrm {T}})$ with the charged particle spectra over the range $0 < p_{\mathrm {T}}< 3$ GeV. *Error bars* show statistical and systematic uncertainties added in quadrature

The large acceptance in $\eta$ of the ATLAS and CMS experiments makes it possible to study whether the observation of the extended longitudinal scaling of $v_2$, when viewed in the rest frame of one of the colliding nuclei, reported by the PHOBOS experiment at RHIC [6, 32], holds at the much higher LHC energy. The PHOBOS measurements of elliptic flow over a range of Au+Au collision energies, $\sqrt{s_{\mathrm {NN}}} = 19.6$, 62.4, 130 and 200 GeV, showed energy independence of the integrated $v_2$ as a function of $|\eta |-y_{{\mathrm {beam}}}$, where $y_{{\mathrm {beam}}}=\ln {(\sqrt{s_{\mathrm {NN}}}/m)}$ is the beam rapidity and $m$ is the proton mass. A similar effect was also observed in charged-particle densities [6] and is known as limiting fragmentation [33]. In Fig. 12, the integrated $v_2$ is plotted as a function of $|\eta |-y_{{\mathrm {beam}}}$ and compared to the PHOBOS results for three centrality bins matching those used by PHOBOS. The PHOBOS results are obtained with the event-plane method for charged particles with a low-$p_{\mathrm {T}}$ limit of 0.035 GeV at mid-rapidity and of 0.004 GeV around the beam rapidity [34]. The CMS data [20] obtained with the event-plane method are also shown. The CMS measurement represents $v_2$ integrated over $p_{\mathrm {T}}$ from 0 to 3 GeV. This measurement was obtained by extrapolating $v_2(p_{\mathrm {T}})$ measured for $p_{\mathrm {T}}> 0.3$ GeV and the charged-particle spectra down to $p_{\mathrm {T}}=0$ under the assumption that $v_2(p_{\mathrm {T}}=0)=0$ and with the charged-particle yield constrained by the measured ${\mathrm {d}}N_{\mathrm {ch}}/{\mathrm {d}}\eta$ distribution [35]. The ATLAS and CMS results agree within the uncertainties, although the CMS $v_2$ is systematically smaller by about 5 % than the ATLAS measurement. This small systematic difference can be attributed to the uncertainty in the CMS extrapolation to $p_{\mathrm {T}}=0$ or the $p_{\mathrm {T}}$ threshold of 0.07 GeV for the ATLAS measurement, or the combination of both.

As can be seen from the figure, there is no overlap in $|\eta |-y_{{\mathrm {beam}}}$ between the PHOBOS and LHC data, so a direct comparison with the low-energy data is not possible. Nevertheless, it can be concluded, keeping in mind the relatively large uncertainties in the low-energy results, that the extrapolation of the trend observed at RHIC to the LHC energy appears to be consistent with the LHC measurements, although the dependence on $|\eta |-y_{{\mathrm {beam}}}$ may be weaker at the LHC energy.

## Summary and conclusions

Measurements of the integrated elliptic flow of charged particles in Pb+Pb collisions at $\sqrt{s_{\mathrm {NN}}} = 2.76$ TeV are presented by the ATLAS experiment at the LHC. The elliptic anisotropy parameter $v_2$ is measured with the event-plane method over a broad range of collision centralities (0–80 %). The kinematic range in pseudorapidity extends out to $|\eta |=2.5$, and in $p_{\mathrm {T}}$ down to 0.07 GeV. This low-$p_{\mathrm {T}}$ region is reached by using a tracklet reconstruction algorithm to analyze about $1~\mu \hbox {b}^{-1}$ of data taken with the solenoid field turned off. Other track reconstruction methods with low-$p_{\mathrm {T}}$ thresholds of 0.1 and 0.5 GeV respectively, are exploited in order to verify the tracklet measurement and provide results that can be directly compared to other LHC measurements. The value of $v_2$ integrated from $p_{\mathrm {T}}= 0.07~\hbox {GeV}~$ provides a reliable estimate of the elliptic flow measured over the range $p_{\mathrm {T}}\ge 0$.

The $p_{\mathrm {T}}$-integrated elliptic flow as a function of collision centrality shows a clear dependence on $p_{\mathrm {T,0}}$, both within the present measurements and in comparison to the CMS results obtained with higher low-$p_{\mathrm {T}}$ limits. The integrated elliptic flow increases with centrality, reaching a maximum of 0.08 for mid-central collisions (40–50 %) and then decreases to about 0.02 for the most central collisions.

The pseudorapidity dependence of the $p_{\mathrm {T}}$-integrated $v_2$ is very weak, with a slight decrease in $v_2$ as $|\eta |$ increases. The results are in agreement with the CMS measurements covering the same $\eta$ range, provided the same low-$p_{\mathrm {T}}$ cutoff is used. The integrated $v_2$ transformed to the rest frame of one of the colliding nuclei is compared to the lower-energy RHIC data. Although a direct comparison is not possible due to the non-overlapping kinematic regions, the general trend observed in the RHIC energy regime seems consistent with the LHC measurements, while the latter may have a weaker dependence on pseudorapidity.

## Figures and Tables

**Fig. 13:**
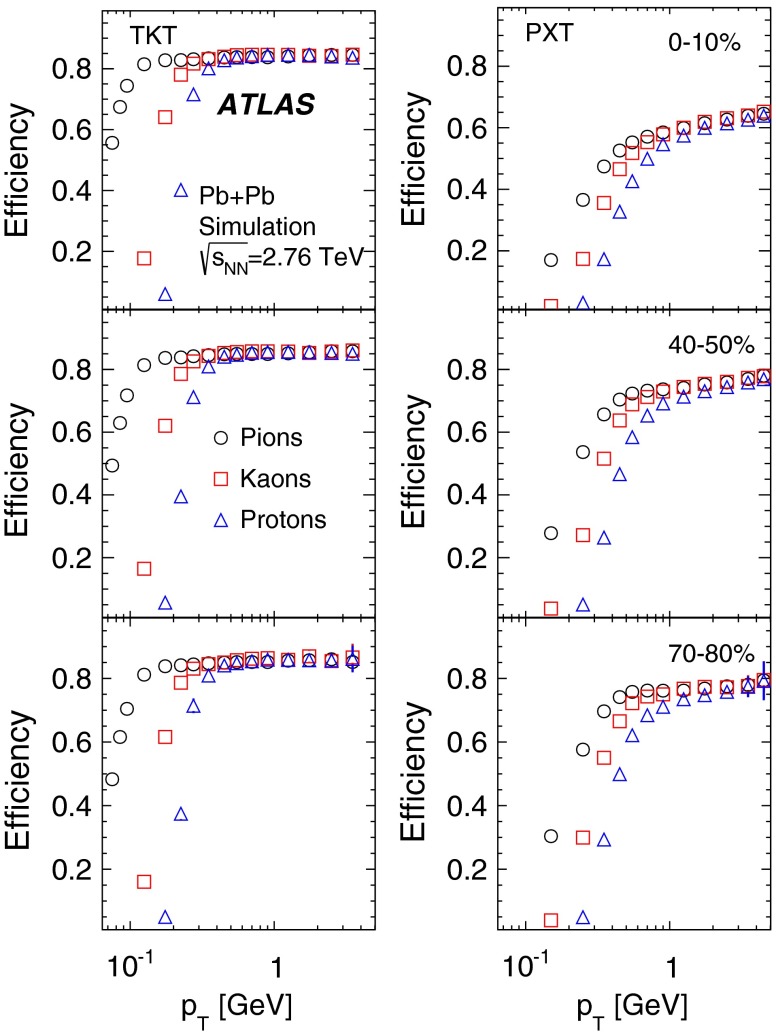
The transverse momentum, $p_{\mathrm {T}}$, dependence of the TKT (*left*) and PXT (*right*) track reconstruction efficiency for $\pi ^{\pm }$, $K^{\pm }$ and $p^{\pm }$ in the pseudorapidity range $|\eta |<1$ for three centrality intervals, as indicated in the legend

## Appendix

In the low-$p_{\mathrm {T}}$ region, the track reconstruction efficiency depends strongly on the particle type. This information is important for comparison of measurements with theory predictions in which the elliptic flow depends on the particle type.

The efficiency of the PXT and TKT methods in reconstructing tracks with $|\eta |<1$ generated as $\pi ^\pm$, $K^\pm$, $p$, and $\bar{p}$ in MC simulation is shown in Fig. 13 as a function of $p_{\mathrm {T}}$. Large differences in efficiency are observed for the PXT method at $p_{\mathrm {T}}$ below about 1 GeV and for the TKT method at $p_{\mathrm {T}}$ below about 0.4 GeV. Above these values, the reconstruction efficiency is independent of particle type. The efficiency is lowest for $p$ and ${\bar{p}}$. For the TKT method, which is most relevant at low $p_{\mathrm {T}}$, the efficiency for reconstructing protons drops to zero below 0.2 GeV.

## Footnotes

1 ATLAS uses the system of units where $c=\hbar =1$.

2 ATLAS uses a right-handed coordinate system with its origin at the nominal interaction point (IP) in the centre of the detector and the $z$-axis along the beam pipe. The $x$-axis points from the IP to the centre of the LHC ring, and the $y$-axis points upward. Cylindrical coordinates $(r,\phi )$ are used in the transverse plane, $\phi$ being the azimuthal angle around the beam pipe. The pseudorapidity is defined in terms of the polar angle $\theta$ as $\eta =-\ln \tan (\theta /2)$.
